# Acupuncture mediates the “gut–testis axis” to improve asthenozoospermia

**DOI:** 10.3389/fendo.2025.1514010

**Published:** 2025-01-27

**Authors:** Jianheng Hao, Huichao Xu, Boya Chang, Jia Ren, Haijun Wang, Laixi Ji

**Affiliations:** ^1^ School of Acupuncture-Moxibustion and Tuina, Chengdu University of Traditional Chinese Medicine, Chengdu, China; ^2^ The Second Clinical College, Shanxi University of Chinese Medicine, Jinzhong, China

**Keywords:** acupuncture, asthenozoospermia, metabolomics, gut microbiota, gut-testis axis

## Abstract

**Background:**

Asthenozoospermia is a common cause of male infertility. Studies have shown that sperm quality and motility are affected by the gut–testis axis that can regulate testicular metabolism and function through the gut microbiota and its metabolites. Acupuncture is an important modality of complementary and alternative medicine. It can improve sperm motility, but it remains unclear whether acupuncture can enhance sperm vitality by influencing the gut–testis axis.

**Methods:**

In this study, sperm quality, testicular pathology, and serum hormone levels were assessed using a cyclophosphamide-induced mouse model. Real-time PCR, a western blot analysis, and immunofluorescence techniques were used to assess the effects of acupuncture on the gut barrier and blood–testis barrier functions. In addition, gut microbiome and metabolomics were used to study the impact of acupuncture on the gut microbiota structure, serum, and testicular metabolites in asthenozoospermic mice. Further validation was obtained by performing a fecal microbiota transplantation (FMT).

**Results:**

Acupuncture improved the sperm quality; ameliorated testicular pathology; increased serum testosterone (T), follicle-stimulating hormone (FSH), and luteinizing hormone (LH) levels; and repaired gut and blood–testis barrier damage in asthenozoospermic mice. The abundances of Bacteroidota, Firmicutes, Faecalibaculum, and Dubosiella were associated with sperm motility, as shown by a gut microbiome analysis. Serum metabolomics revealed that differentially expressed metabolites (DEMs), such as cytosine and N-oleyl-leucine, were closely related to sperm motility. Testicular metabolomics analysis revealed DEMs, such as 5-fluorouridine and 1-acetylimidazole, were also associated with sperm motility. Furthermore, reproductive function improvements in asthenozoospermic mice through acupuncture were achieved via an FMT.

**Conclusion:**

Acupuncture may alleviate asthenozoospermia symptoms by modulating the gut–testis axis and repairing the gut–testis barrier.

## Introduction

Infertility issues are experienced by approximately 15% of couples of reproductive age worldwide, with male fertility issues accounting for approximately 50% of these cases (1). Decreased sperm quality is the most prominent manifestation of male infertility, and reduced sperm motility is particularly prominent. Sperm motility is a crucial male infertility clinical indicator, as only motile sperm can navigate through the female reproductive tract to reach the fertilization site. According to the World Health Organization (WHO) manual for human semen analysis, asthenozoospermia is a condition where after two to seven days of abstinence and at least two semen analyses, the progressively motile sperm proportion is less than 32% or the total sperm motility is less than 40% (2). This is a complex, multifactorial disease that is associated with sperm structure and energy deficiency abnormalities caused by factors such as flagellar defects, mitochondrial dysfunction, oxidative stress, and genetic anomalies (3–6). Common asthenozoospermia treatments include western medications such as L-carnitine and vitamin E. For severe cases, assisted reproductive technologies, such as *in vitro* fertilization (IVF) or intracytoplasmic sperm injection (ICSI), are viable options (7–9). However, the current treatments have limited efficacy and numerous side effects for some patients, and there is a growing interest in exploring traditional Chinese medicine (TCM) as a complementary or alternative therapeutic approach.

Acupuncture is an important TCM component that has shown significant efficacy in treating male infertility conditions such as asthenozoospermia. The testicular microenvironment may be improved through acupuncture as the technique can regulate the hypothalamic–pituitary–gonadal axis, enhance the local blood supply, reduce oxidative stress damage, and alleviate inflammatory responses, thereby increasing both sperm quantity and quality (10–12). The “Zhibian (BL 54)-to-Shuidao (ST 28)” acupuncture technique is derived from the Inner Canon of the Yellow Emperor. Our research team has recently used extensive clinical trials and anatomical studies of acupuncture points on cadavers to establish a comprehensive acupuncture system. We conducted previous studies that have consistently demonstrated that this acupuncture method effectively stimulates the pelvic plexus and pudendal nerve and has demonstrated significant therapeutic advantages for various male and female urinary and reproductive system diseases (13–16).

The “gut–testis axis” concept has garnered attention in recent years, and research has indicated a bidirectional relationship between the gut microbiota and testicular function (17–19). The gut microbiota can modulate androgen and other hormone levels, influencing testicular hormone secretion and spermatogenesis. In addition, the gut microbiota can regulate testicular immune responses and the metabolic environment through immune modulation and metabolic byproducts, thereby maintaining the blood–testis barrier (BTB) integrity and supporting normal sperm development. Gut microbiota dysbiosis may also lead to oxidative stress that can deteriorate sperm quality. Conversely, androgens and other signaling factors released by the testes can also influence the gut microbiota composition. Thus, the gut–testis axis plays a significant role in male reproductive health. However, it remains unclear whether acupuncture can improve sperm motility by modulating the “gut–testis axis." Therefore, the aim of this study is to analyze the impact of acupuncture on the gut microbiome structure and metabolite composition in a mouse model of asthenozoospermia. We will use gut microbiomics and metabolomics to explore the potential mechanism by which the “Zhibian (BL 54)-to-Shuidao (ST 28)” acupuncture technique regulates the gut–testis barrier to alleviate asthenozoospermia.

## Materials and methods

### Experimental animal

Male-specific pathogen-free (SPF) C57BL/6 mice (6−7 weeks old, 18 ± 1 g) were obtained from the Sibeifu Animal Company (SCXK(JING)-2019-0010, Beijing, China) and housed at the Animal Experimental Center of the Shanxi University of TCM. They were placed in a controlled environment (25 ± 2°C, 55 ± 5% humidity, 12-h light/dark cycle) with free access to food and water. They were acclimated for one week prior to the experiment. All animal procedures followed the National Institute of Health (NIH) guidelines and were approved by the Shanxi University of Chinese Medicine Animal Ethics Committee (No. AWE202303381).

### Grouping and modeling

Mice were randomly divided into control, model, and acupuncture (ACU) groups with 14 mice per group. This was accomplished using a random number table. The model and ACU groups received intraperitoneal injections of cyclophosphamide (HY-17420, MedChemExpress, NJ, USA) at 50 mg/kg/day to induce an asthenozoospermia model (20), while the control group received an equivalent volume of 0.9% sodium chloride solution that was administered for five consecutive days. The ACU group mice underwent acupuncture treatment for two weeks one day after model induction. Disposable acupuncture needles (0.25 mm×13 mm) were inserted bilaterally at the “Zhibian (BL 54)” points (located in the bony fissure where the line between the greater trochanter and the midpoint of the fourth sacral vertebra meets) and angled toward the “Shuidao (ST 28)” points (approximately 8 mm below the junction of the upper 2/3 and lower 1/3 between the xiphoid process and pubic symphysis and 3 mm lateral to the midline) (16) at a depth of 5 mm for 20 min each session once daily. The control and model group mice remained fixed without further interventions. The mice were anesthetized with isoflurane after the treatments, and samples were collected for subsequent experiments.

### Fecal microbiota transplantation experiment

A total of 12 mice were randomly divided into two groups (FMT-M and FMT-A). A cyclophosphamide injection (50 mg/kg/d) was first administered intraperitoneally for five consecutive days to induce asthenozoospermia in the mice. Gavage was then used to administer an antibiotic mixture daily for seven days to clear the existing gut microbiota (21). The antibiotic mixture consisted of vancomycin (100 mg/kg), neomycin sulfate (200 mg/kg), metronidazole (200 mg/kg), and ampicillin (200 mg/kg) (MB1260, MB1716, MB2200, and MB1378 were purchased from Meilun Biological, Dalian, China). Model and ACU group fecal samples that were collected in a previous experiment were homogenized in a 0.1 g feces/1 mL phosphate buffer solution (PBS) ratio, and the supernatants were collected using centrifugation at 100 g for 5 min at 4°C. The model and ACU group supernatants were then transferred to the FMT-M and FMT-A mice groups at a dose of 10 mL/kg/d via gavage for seven consecutive days.

### Determination of the testicular mass and coefficient

All mice in each group were placed into a specialized small animal inhalation anesthesia machine and anesthetized using 2.5% isoflurane. The mice were then placed in a supine position and fixed on a dissection table. The abdominal skin was disinfected with alcohol, and the abdominal skin and muscles were carefully cut along the midline using scissors and forceps to expose the abdominal cavity. The testes and epididymal tissues were gently lifted with forceps and carefully separated from surrounding blood vessels, vas deferens, and other tissues. Excess fat tissue was trimmed off, and the tissues were placed on filter paper to absorb excess moisture. An electronic balance was used to weigh the testes, and the formula for the testicular coefficient (%) was mass of both testes (g)/final body weight of the mouse (g) × 100%.

### Sperm quality determination

All mice in each group were anesthetized, and the abdominal skin was disinfected with alcohol. The midline was cut using scissors and forceps to open the abdominal skin and muscle and expose the abdominal cavity. The epididymal tissue was excised to remove any surrounding adipose tissue. The tissues were then cut into small pieces, placed in a petri dish that contained 1 mL of physiological saline, and incubated in a 37°C water bath for 30 min to allow the sperm to be fully released. The supernatant was collected to obtain the epididymal sperm. A total of 20 μL of sperm suspension was placed using a pipette onto a pre-warmed sperm counting chamber at 37°C. This was then analyzed using a computer-assisted sperm analysis (CASA) system (WLJY-9000, Weili Co., Beijing, China). Ten fields of each sample were examined to determine the sperm concentration, viability, and motility (22).

### Serum testosterone, follicle-stimulating hormone, and luteinizing hormone concentration determinations

Blood samples were collected using the eyeball removal method. The blood was allowed to stand for 2 h, and it was then centrifuged at 3500 r/min for 15 min at 4°C (centrifuge radius 8.2 cm). The upper serum was collected and stored at –80°C for later analysis. An enzyme-linked immunosorbent assay (ELISA) detection was performed using seven random serum samples from each group. A total of 100 μL of a standard solution was added to each blank and standard well, while 100 μL of the sample was added to each sample well. A pipette was used to thoroughly mix the mixture, and it was incubated at 37°C for 90 min. The liquid was then discarded, and 100 μL of a biotinylated antibody working solution was added to each well for a 1-h incubation at 37°C. The plate was then washed, and the excess liquid was removed (repeated three times). A total of 100 μL of an enzyme conjugate working solution was then added to each well, and the wells were incubated for 30 min at 37°C. The washing step was repeated five times. Next, 90 μL of a substrate solution was added to each well. The plate was then incubated at 37°C in the dark for 15 min. Finally, 50 μL of a stop solution was added to terminate the reaction. A microplate reader (Spectra Max Plus384, Molecular Devices, Sunnyvale, USA) was used to measure the absorbance at 450 nm, and the testosterone (T), follicle-stimulating hormone (FSH), and luteinizing hormone (LH) concentrations were calculated (MU30398, MU30265, MU30382, Bio-swamp, Wuhan, China).

### H&E staining

Seven unilateral testicular tissues from each group were randomly selected and fixed in a testicular fixative (G1121, Servicebio, Wuhan, China) for 24 h. The tissues were then transferred to 75% ethanol for 48 h. In addition, seven colon tissues from each group were randomly selected and fixed in a formaldehyde solution for 24 h. Paraffin sections that were 5 μm (RM 2145, LEICA, Wetzlar, Germany) were prepared after dehydration, clearing, paraffin infiltration, and embedding. The paraffin sections were dehydrated using a gradient of 85% and 95% ethanol and then stained with hematoxylin for 3 min. This was followed by eosin staining for 5 min. The sections were dried and mounted with neutral resin and examined under a microscope (ECLIPSE Ci-S, Nikon, Tokyo, Japan) to investigate the testicular and colon tissue histopathological changes. Three different sections were observed for each mouse.

### Diff-Quik staining

Three epididymal tissue samples were randomly selected from each group and placed in a culture dish. A total of 1 mL of a 0.9% sodium chloride solution was then added. A sterile disposable needle was used to gently puncture the head of the epididymis to release sperm into the culture dish. An appropriate amount of sperm suspension was then evenly pipetted onto a glass slide that was subsequently placed in Dif Quick fixative (G2572, Solarbio, Beijing, China) for 15−20 s. The slide was then stained sequentially in Dif Quick Staining Solution I and II for 5−10 s each. This was followed by 10−15 washes in running water to remove excess dye. After drying, the sperm morphology was observed under a microscope.

### Real-time PCR analysis

The total RNA was extracted from the colon samples of each group and reverse transcribed to synthesize the cDNA. This cDNA was used as a template for PCR amplification under the following conditions: 95°C for 30 s (1 cycle); 95°C for 15 s; and 60°C for 30 s (40 cycles). The Ct values of each well after the reaction were recorded using a PCR machine (CFX 96, Bio-Rad, CA, USA). Glyceraldehyde 3-phosphate dehydrogenase (GAPDH) was used as an internal control, and the relative expression levels of the target genes were calculated using the 2^–△△CT^ method. The primer sequences are listed in **Table 1** (designed by Servicebio, Wuhan, China).

**Table 1:** Primer sequences.

Gene	Primer sequences (5’–3’)	Amplicon size (bp)
Occludin	Forward: CACCTCCTTACAGACCTGATGAAT	318
	Reverse: AGCCACCTCCGTAGCCAAA	
ZO-1	Forward: GGGAAAACCCGAAACTGATG	103
	Reverse: GCTGTACTGTGAGGGCAACG	
Claudin-1	Forward: GTGTCCTACTTTCCTGCTCCTGT	101
	Reverse: TCACACATAGTCTTTCCCACTAGAAG	
GAPDH	Forward: CCTCGTCCCGTAGACAAAATG	133
	Reverse: TGAGGTCAATGAAGGGGTCGT	

### Western blot analysis

The total protein was extracted from the testicular and colonic tissues of each mouse group. The bicinchoninic acid assay (BCA) method was used to determine the protein concentrations. Protein samples were loaded into a gel for electrophoresis. This was followed by wet transfer and blocking for 2 h each. The bands were then placed in separate wet chambers, and each chamber was incubated overnight at 4°C with the following antibodies: ZO-1 (A0659, Abclonal, Wuhan, China), Occludin (GB111401, Servicebio, Wuhan, China), Claudin-1 (GB112543, Servicebio, Wuhan, China), Connexin-43 (A11752, Abclonal, Wuhan, China), and N-cadherin (A3045, Abclonal, Wuhan, China). The dilution ratios were 1:1000 for ZO-1, Occludin, Claudin-1, Connexin-43, and N-cadherin antibodies, and 1:3000 for β-actin. The membranes were washed the following day with tris-buffered saline and Polysorbate 20 (TBST) and incubated with the secondary antibody (dilution 1:5000) for 2 h. The membranes were then washed again with TBST, and a chemiluminescent detection solution was added for imaging. The relative expression levels of the target proteins among the different samples were analyzed by comparing the gray values of the bands using Image J software (Bethesda, Maryland, USA).

### Detection and analysis of the fecal intestinal microbiota

Seven mice were randomly selected from each group. An iodine tincture was used to disinfect the perianal skin of the mice in the morning following the treatment. The mice were then gently held in a head-up position with their body suspended, and their abdomen was massaged to help them pass fresh feces. Four to five fecal pellets were collected from each mouse and placed into a sterile centrifuge tube. The samples were quickly transferred to liquid nitrogen for 3 h, and these samples were then stored at –80°C for later use. A DNA extraction kit (D5635-02, BioTek, Norcross, GA, USA) was used to extract the microbial DNA from the fecal samples. Amplification and sequencing were performed using gene-specific primers AMV4.5N (5′-AAGCTCGTAGTTGAATTTCG-3′) and AMDGR (5′-CCCAACTATCCCTATT AATCAT-3′). The PCR products were purified using the AxyPrep DNA Gel Extraction Kit (Axygen Biosciences, Union City, CA, USA) and quantified using the Quant-iT PicoGreen dsDNA Assay Kit (P7589, Thermo Fisher, Waltham, MA, USA) on a microplate reader (FLx800, BioTek, Norcross, GA, USA). The Illumina NovaSeq platform (NovaSeq 6000, Illumina, San Diego, CA, USA) was used to perform the sequencing with assistance from the Shanghai Personal Biotechnology Co., Ltd. Bioinformatics tools were then used to analyze the raw sequencing data that included the sequence quality control, sequence alignment, operation taxonomic unit (OTU) clustering, and classification steps to determine the microbial species present in the samples and their relative abundances.

### Detection and analysis of serum and testicular metabolomics

Testicular and serum samples were randomly collected from seven mice from each group. Homogenization beads and 500 μL of the extraction solvent (methanol:acetonitrile:water = 2:2:1) were added to the samples. After grinding and centrifugation, the supernatant was transferred to sample vials for analysis using ultra-high performance liquid chromatography with quadrupole time-of-flight mass spectrometry (UPLC-Q-TOF/MS) (Vanquish, Thermo Fisher Scientific, Waltham, MA, USA). The chromatographic conditions included an ACQUITY UPLC^®^ HSS T3 column (150 mm × 2.1 mm, 1.8 μm) with a mobile phase of 0.1% formic acid in water (A) and 0.1% formic acid in acetonitrile (B) at a flow rate of 0.35 mL·min^–1^, a column temperature of 50°C, and an injection volume of 5 μL. Gradient elution was performed for positive ions (0–1 min, 5% B; 1–24 min, 5%–100% B; 24–28 min, 100% B; 28–30 min, 100%–5% B; and 30–33 min, 5% B) and negative ions (0–1 min, 2% B; 1–18 min, 5%–100% B; 18–22 min, 100% B; 22–25 min, 100%–2% B; and 25–28 min, 2% B). Mass spectrometry conditions included an electrospray ionization (ESI) source with ion source temperatures of 350°C (positive ion mode) and 300°C (negative ion mode), a capillary temperature of 320°C, a sheath gas flow rate of 35 psi, and an auxiliary gas flow rate of 10 psi. The spray voltages were 3.5 kV for positive ions and 2.5 kV for negative ions. The full scan resolution was 70,000 full width at half maximum (FWHM), the secondary scan resolution was 17,500 FWHM, the mass detection range was m/z 100–1,500, the dynamic exclusion duration was 8 s, and the collision energies were 20, 40, and 60 eV. The raw data were then processed and normalized, and the orthogonal partial least squares discriminant analysis (OPLS-DA) model was used to obtain the variable importance for projection (VIP) values > 1 and P values < 0.05 as criteria for the selection of significant differential metabolites (DEMs). DEM screening was conducted in the combined ESI+ and ESI– modes, with overlapping substances between different modes retained based on the larger VIP value. A functional analysis was then performed on the DEMs.

### Immunofluorometric assay

The paraffin-embedded testis tissue sections were first dewaxed to water. The sections were then placed in a retrieval box filled with an ethylenediaminetetraacetic acid (EDTA) antigen retrieval buffer (G1203, Servicebio, Wuhan, China). The sections were then subjected to antigen retrieval in a microwave. The slides were washed in PBS (G0002, Servicebio, Wuhan, China) on a decolorizing shaker for 3×5 min after being naturally cooled. The sections were then slightly dried, and a circle was drawn around the tissue using a histochemical pen. Bovine serum albumin (BSA) (GC305010, Servicebio, Wuhan, China) was added to the circle for a 30 min incubation. The blocking solution was gently removed, and the primary antibodies (Connexin-43 and N-cadherin at 1:200 dilution, Occludin at 1:1000 dilution) were added. The sections were then placed flat in a humidified chamber and incubated overnight at 4°C. After being returned to room temperature the following day, the slides were washed in PBS for 3×5 min. The secondary antibody (GB21303, Servicebio, Wuhan, China) was added and incubated for 50 min at a 1:300 dilution. The sections were then slightly dried, and a 4′,6-diamidino-2-phenylindole (DAPI) staining solution (G1012, Servicebio, Wuhan, China) was added within the circle. This was then incubated in the dark at room temperature for 10 min. The slides were then mounted, and the images were observed under a fluorescence microscope.

### Statistical analysis

GraphPad Prism 7 software was used for the data analysis. Data that followed a normal distribution are presented as means ± standard deviation (SD), and differences between the two groups were analyzed using Student’s t-test. Differences among multiple groups were assessed using a one-way analysis of variance (ANOVA). This was followed by a *post-hoc* least significant difference (LSD) test. The relationships between the gut microbiota and metabolomics variables were explored using Spearman correlation analyses.

## Results

### Acupuncture improved the testicular function and sperm quality in the asthenozoospermic mice

**Figure 1:**
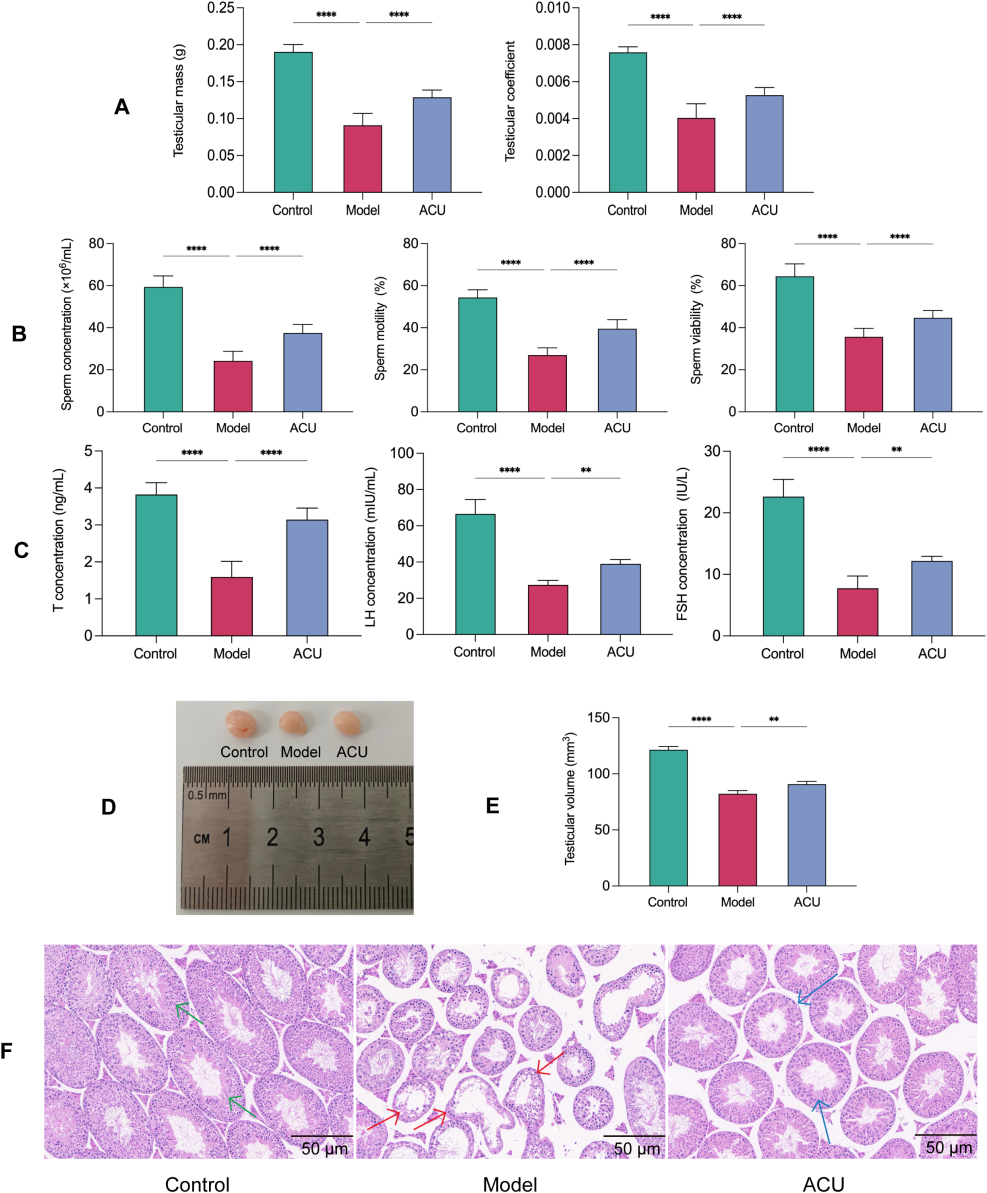
Acupuncture improved the testicular function and sperm quality in the asthenozoospermic mice. **(A)** Comparison of testicular mass and testicular coefficient of each group (n=14). **(B)** Comparison of sperm parameters of each group (n=14). **(C)** Comparison of serum T, FSH, and LH concentrations of each group (n=7). **(D)** Comparison of visual observation of testicles of each group. **(E)** Comparison of testicular volume of each group (n=7). **(F)** Comparison of testicular histopathological morphology of each group (n=7). Green arrows indicate seminiferous tubules with normal morphology and structure, red arrows indicate tubules with uneven wall thickness and dilated lumens, and blue arrows indicate tubules with improved morphology and structure. Data are presented as mean ± SD. **P < 0.01, ****P < 0.0001.

The key indicators for improving symptoms of asthenozoospermia are the testicular mass, the testicular coefficient, and the sperm quality. The model group exhibited significant declines in the testicular mass, the testicular coefficient, the sperm concentration, the sperm motility, and the sperm viability (*P* < 0.01) compared with the control group. These parameters showed varying degrees of improvement (*P* < 0.01) after acupuncture treatment (**Figures 1A, B**). Asthenozoospermia is often associated with imbalances in serum hormone levels. In this study, the model group had reduced T, FSH, and LH serum levels compared with the control group (*P* < 0.01), while the ACU group showed varying degrees of increase in these hormone levels compared with the model group (*P* < 0.01) (**Figure 1C**). Sperm production primarily occurs in the testis and any structural or functional abnormalities can impact the processes of spermatogenesis and sperm maturation. We observed that the testicular volume in the model group mice decreased (*P* < 0.01) and had uneven coloration, a roughened surface, and abnormal vascular distribution in some areas (**Figures 1D, E**). H&E staining in the model group revealed disorganized seminiferous tubule structures, deformed or collapsed lumens, reduced or disordered arrangement of spermatogenic cells, and decreased sperm counts (**Figure 1F**). In addition, the interstitial tissue showed signs of widening or edema. Both the testicular volume and pathological morphology of the mice showed varying degrees of improvement after acupuncture treatment. These results indicated that acupuncture effectively enhanced the testicular function and sperm quality in the asthenozoospermic mice.

### Acupuncture regulated the gut microbiota structure in the asthenozoospermic mice

**Figure 2:**
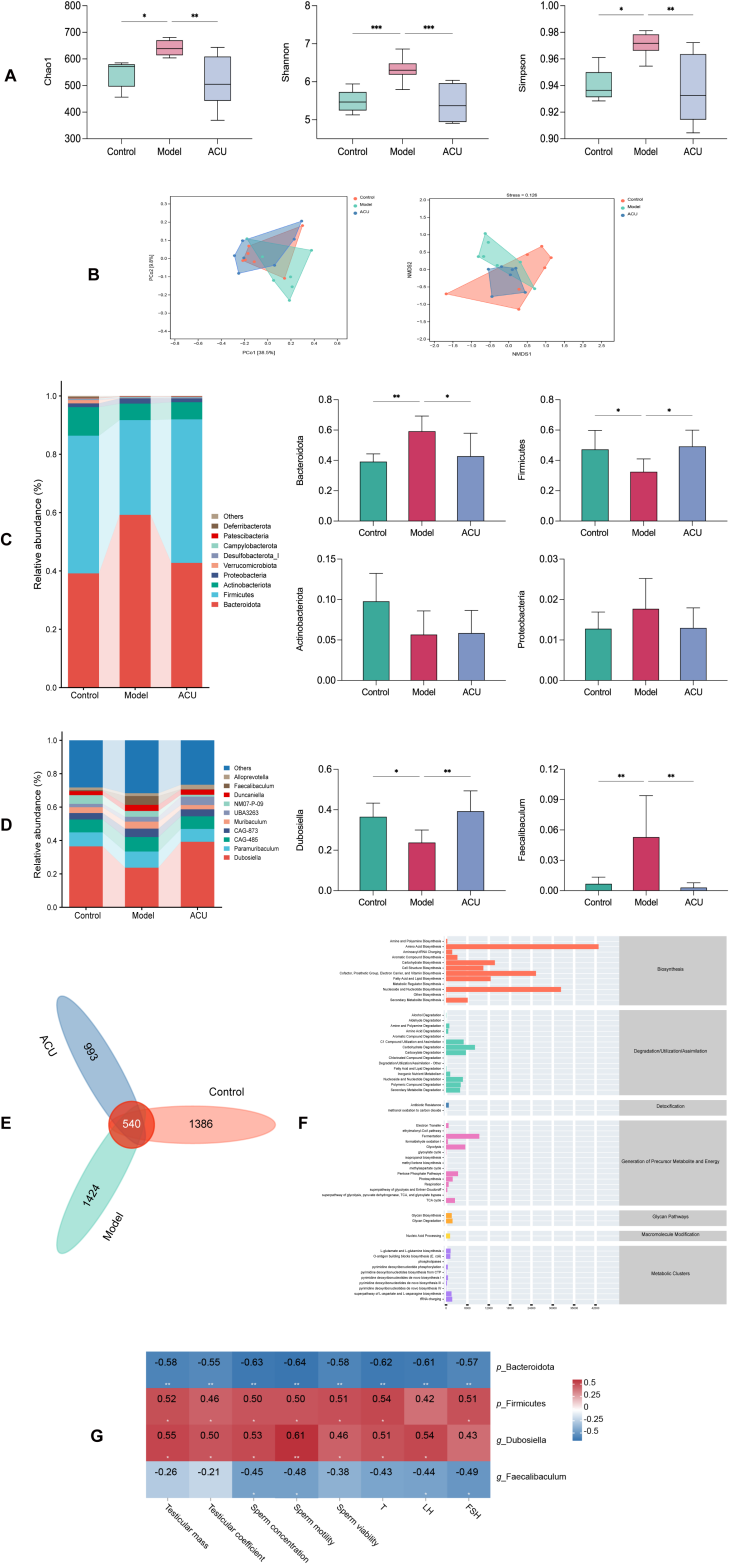
Acupuncture regulated the gut microbiota structure in the asthenozoospermic mice. **(A)** Comparison of α-diversity indices of each group. **(B)** Comparison of β-diversity of each group. **(C)** Comparison of differences at the phylum level of each group. **(D)** Comparison of differences at the genus level of each group. **(E)** Venn diagram of shared species. **(F)** Predicted metabolic pathways of shared species. **(G)** Spearman correlation analysis. Data are presented as mean ± SD. *P < 0.05, **P < 0.01, ***P < 0.001.

The gut microbiota is involved in various male reproductive physiological processes, and it can affect sperm quality (23). Therefore, the gut microbiota changes between the different groups were analyzed. The α-diversity index primarily represents richness using the Chao1 index and diversity using the Shannon and Simpson indices. The model group showed significant increases in the Chao1, Shannon, and Simpson indices compared with the control group (*P* < 0.01, *P* < 0.05). The ACU group showed significant decreases in the Chao1, Shannon, and Simpson indices compared with the model group (*P* < 0.01). These results both indicated that the gut microbiota richness and diversity decreased after acupuncture (**Figure 2A**). A principal coordinate analysis (PCoA) and nonmetric multidimensional scaling (NMDS) were used to perform the dimensionality reduction on the multidimensional microbial data to analyze the differences in the β-diversity between samples (**Figure 2B**). The control group and the model group were more dispersedly clustered in the PCoA plot. This result suggested significant changes in the gut microbiota structure between the two groups. The ACU group clustered closer to the control group and further from the model group. This indicated a regulatory effect of acupuncture on the gut microbiota. The NMDS stress value (stress) was 0.126 < 0.2, indicating that the study results were relatively reliable.

Bacteroidota, Firmicutes, Actinobacteriota, and Proteobacteria were the primary dominant phyla of the gut microbiota of each mice group, and they accounted for greater than 90% of the total phyla (**Figure 2C**). The model group showed a significant increase in the relative abundance of Bacteroidota (*P* < 0.01) and a decrease in the relative abundance of Firmicutes (*P* < 0.05) compared with the control group, while the differences in Actinobacteriota and Proteobacteria were not significant (*P* > 0.05). The ACU group showed a significant decrease in the relative abundance of Bacteroidota (*P* < 0.05) and an increase in the relative abundance of Firmicutes (*P* < 0.05) compared with the model group. The dominant genera in the gut microbiota of each group were *Dubosiella* and *Paramuribaculum*, accounting for greater than 50% of the total genera (**Figure 2D**). However, only *Dubosiella* and *Faecalibaculum* showed significant differences between the control group and the model group (*P* < 0.01, *P* < 0.05). The relative abundance of *Dubosiella* increased (*P* < 0.01), while the relative abundance of *Faecalibaculum* decreased (*P* < 0.01) after acupuncture. A Venn diagram was plotted to identify a total of 540 shared species (**Figure 2E**). The Kyoto Encyclopedia of Genes and Genomes (KEGG) database was used to predict the metabolic pathways of the intersecting species. It was revealed that the pathways were primarily related to amino acid biosynthesis, nucleoside, and nucleotide biosynthesis (**Figure 2F**). The relationship between the gut microbiota and asthenozoospermia was examined using a Spearman correlation analysis to study the correlation between significantly different *p*_Bacteroidota, *p*_Firmicutes, *g*_*Dubosiella*, and *g*_*Faecalibaculum* with the major parameters of asthenozoospermia (**Figure 2G**). The results showed that *p*_Bacteroidota and *g*_*Faecalibaculum* were negatively correlated with the sperm concentration and motility (*P* < 0.01, *P* < 0.05), while *p*_Firmicutes and *g*_*Dubosiella* were positively correlated with the sperm concentration and motility (*P* < 0.01, *P* < 0.05).

### Acupuncture improved intestinal barrier damage in the asthenozoospermic mice

**Figure 3:**
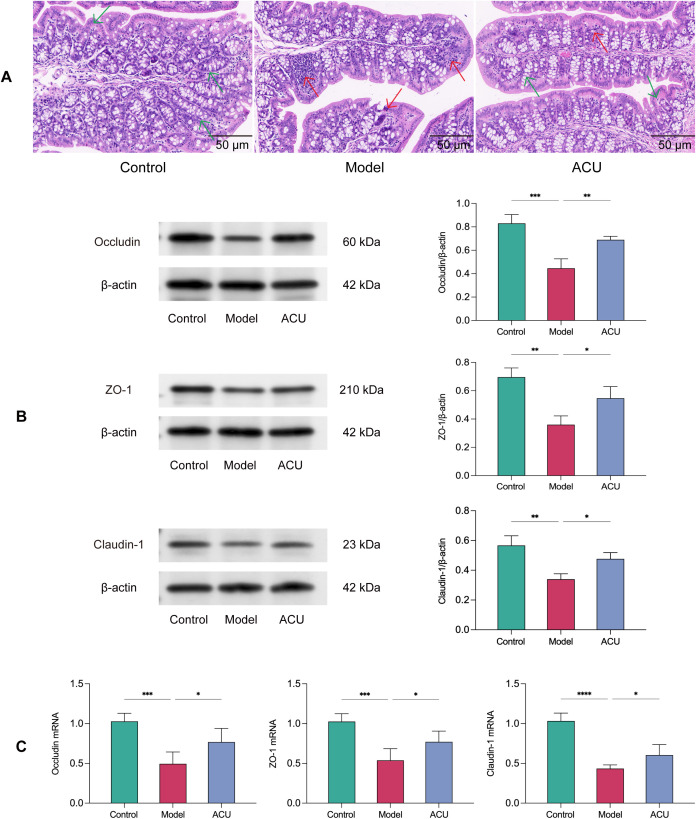
Acupuncture improved intestinal barrier damage in the asthenozoospermic mice. **(A)** Comparison of pathological morphology of colon tissues of each group (n=7). **(B)** Comparison of Occludin, ZO-1, and Claudin-1 protein expression levels in colonic tissues of each group (n=3). **(C)** Comparison of Occludin, ZO-1, and Claudin-1 mRNA expression levels in colonic tissues of each group (n=5). Note: The green arrows indicate morphologically normal colonic tissue, while the red arrows indicate colonic tissue with disrupted intestinal barrier and inflammatory infiltration. Data are presented as mean ± SD. *P < 0.05, **P < 0.01, ***P < 0.001, ****P < 0.0001.

Gut microbiota balance and health are crucial to maintain intestinal barrier integrity. An adverse gut microbiota structure can disrupt the intestinal barrier and cause various diseases (23). The H&E staining revealed that the intestinal barrier in the colonic tissue of the model group mice was disrupted, and this was accompanied by varying degrees of inflammatory infiltration (**Figure 3A**). We further evaluated the protein and mRNA expression levels of Occludin, ZO-1, and Claudin-1 using western blotting and real-time PCR, respectively (**Figures 3B, C**). The results showed that the mRNA and protein expression levels of Occludin, ZO-1, and Claudin-1 were significantly downregulated in the model group (*P* < 0.01, *P* < 0.05). However, these changes were reversed after acupuncture treatment (*P* < 0.01, *P* < 0.05), indicating that acupuncture can improve impaired intestinal barrier function.

### Acupuncture regulated serum metabolic disorders in the asthenozoospermic mice

**Figure 4:**
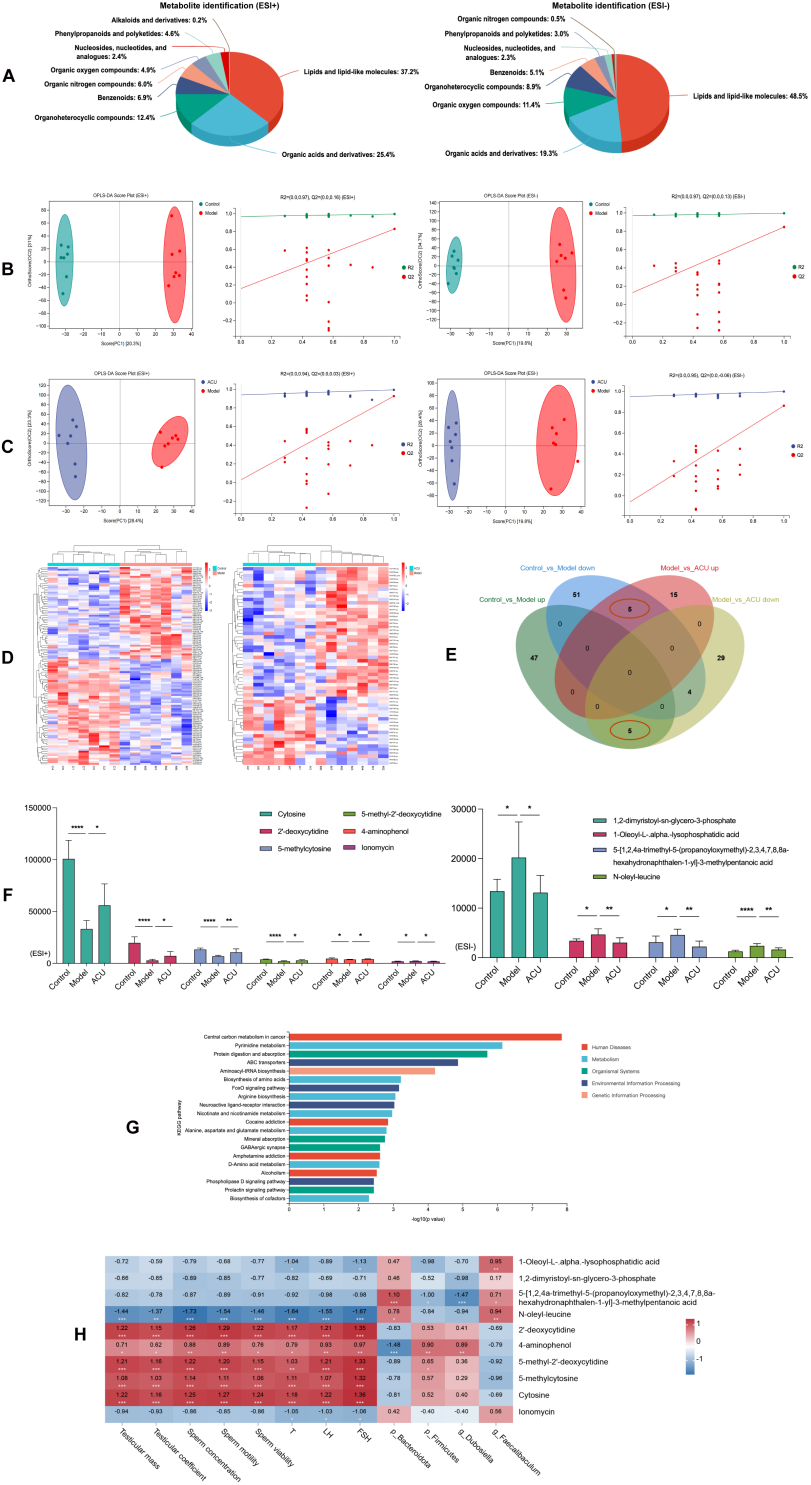
Acupuncture regulated serum metabolic disorders in the asthenozoospermic mice. **(A)** Serum metabolite identification and analysis in ESI+ and ESI- Modes. **(B)** OPLS-DA analysis and permutation test between control and model groups in ESI+ and ESI- Modes. **(C)** OPLS-DA analysis and permutation test between model and ACU groups in ESI+ and ESI- Modes. **(D)** Clustering analysis of metabolites among groups in combined ESI+ and ESI- modes. **(E)** Venn diagram of intersecting differential metabolites in combined ESI+ and ESI- modes. **(F)** Comparison of differential metabolite expression between ESI+ and ESI- modes. **(G)** KEGG Enrichment analysis. **(H)** Spearman correlation analysis. *P < 0.05, **P < 0.01, ***P < 0.001, ****P < 0.0001.

Gut barrier disruption can cause the movement of harmful gut microbes across the barrier into the bloodstream. This triggers inflammatory responses and immune system dysregulation, altering the composition of plasma metabolites (24). Therefore, we analyzed the metabolites in the serum of each group. We performed classification statistics on all identified metabolites based on their chemical classification information. This analysis allowed us to display the proportion of each metabolite category using pie charts. The results indicated that under both ESI+ and ESI– modes, the metabolites were primarily composed of lipids and lipid-like molecules and organic acids and derivatives (**Figure 4A**). OPLS-DA model were then used to detect intergroup differences. **Figures 4B, C** shows the comparisons between the control group and the model group, as well as between the model group and the ACU group. There were significant separations between these groups, while the distribution points within each group were clustered. In addition, the internal cross-validation results of this model indicated that in the ESI+ mode, between the control group and the model group, R2Y = 0.997 and Q2Y = 0.830, and between the model group and the ACU group, R2Y = 0.994 and Q2Y = 0.927. In the ESI- mode, between the control group and the model group, R2Y = 0.996 and Q2Y = 0.846, and between the model group and the ACU group, R2Y = 0.999 and Q2Y = 0.863. These results suggested that the model had strong interpretability and good predictability. We also conducted permutation tests as an external validation method to further validate the model reliability. **Figures 4B, C** also shows that all of the Q2 points were lower than the rightmost original Q2 point. This indicated that the results of this study were reliable and effective.

In the combined ESI+ and ESI– modes, 112 DEMs were identified between the control group and the model group (52 upregulated and 60 downregulated), and 58 DEMs were identified between the model group and the ACU group (20 upregulated and 38 downregulated) (**Supplementary Table 1**). The clustering heatmap showed significant differences between the control group vs. model group and model group vs. ACU group (**Figure 4D**), meeting the criteria for further analysis. A Venn diagram was drawn (**Figure 4E**), and we found that five DEMs were upregulated in the model group and downregulated after acupuncture treatment, while five DEMs were downregulated in the model group and upregulated after acupuncture treatment (**Figure 4F**). These 10 DEMs may be important targets through which acupuncture improves the condition, and they were then used in the subsequent functional enrichment analysis. The KEGG analysis revealed that these DEMs were primarily associated with pathways such as pyrimidine metabolism, protein digestion and absorption, and ABC transporters. (**Figure 4G**). A Spearman correlation analysis was performed to explore the relationships between the 10 DEMs and the key parameters of asthenozoospermia, as well as the gut microbiota (**Figure 4H**). Cytosine, 2’-deoxycytidine, 5-methylcytosine, 5-methyl-2’-deoxycytidine, and 4-aminophenol all showed a positive correlation with the sperm parameters or serum hormone levels (*P* < 0.01, *P* < 0.05). N-oleyl-leucine was negatively correlated with these sperm parameters or serum hormone levels (*P* < 0.01). In addition, several metabolites exhibited significant correlations with the gut microbiota (*P* < 0.01, *P* < 0.05).

### Acupuncture improved BTB damage in the asthenozoospermic mice

**Figure 5:**
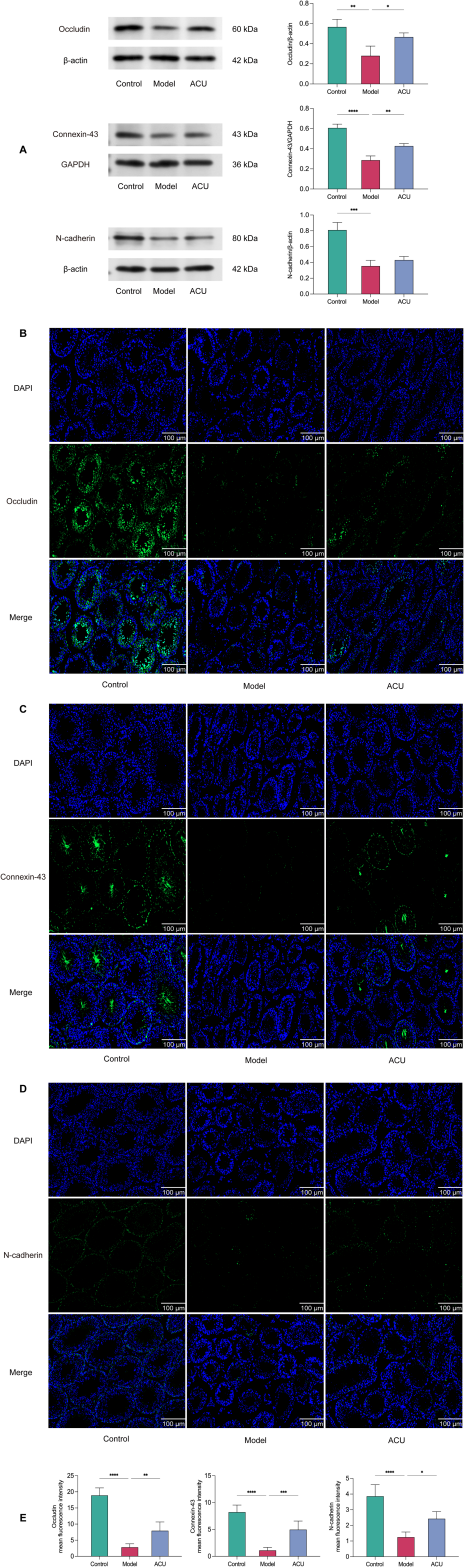
Acupuncture improved BTB damage in the asthenozoospermic mice. **(A)** Comparison of Occludin, Connexin-43, and N-cadherin protein expression levels in the testicular tissues of each group (n=3). **(B-E)** Immunofluorescence comparison of Occludin, Connexin-43, and N-cadherin in the testicular tissues of each group (n=3). Data are presented as mean ± SD. *P < 0.05, **P < 0.01, ***P < 0.001, ****P < 0.0001.

Disruption of the BTB integrity is a significant cause of impaired spermatogenesis (25). We evaluated the expressions of the key BTB proteins, Occludin, Connexin-43, and N-cadherin, using western blotting to investigate the acupuncture effect on BTB in the asthenozoospermic mice (**Figure 5A**). We observed the distribution of these proteins within the testes using immunofluorescence (**Figures 5B–E**). The results showed that the positive signal for the tight junction protein Occludin was primarily expressed at the tight junctions formed by the Sertoli cells, particularly in the BTB region of the seminiferous tubules. Connexin-43 positive signals were detected in the spermatogenic cells, Sertoli cells, and the surrounding interstitial cells of the testes. N-cadherin was primarily expressed in the contact areas between the Sertoli cells and spermatogenic cells. The protein expressions of Occludin, Connexin-43, and N-cadherin were reduced to varying degrees after the cyclophosphamide treatment (*P* < 0.01). This result indicated that the BTB integrity was compromised. However, acupuncture treatment mitigated the reduction trend in the protein levels of Occludin and Connexin-43 (*P* < 0.01, *P* < 0.05), suggesting that acupuncture can protect BTB integrity.

### Acupuncture regulated testicular metabolic disorders in the asthenozoospermic mice

**Figure 6:**
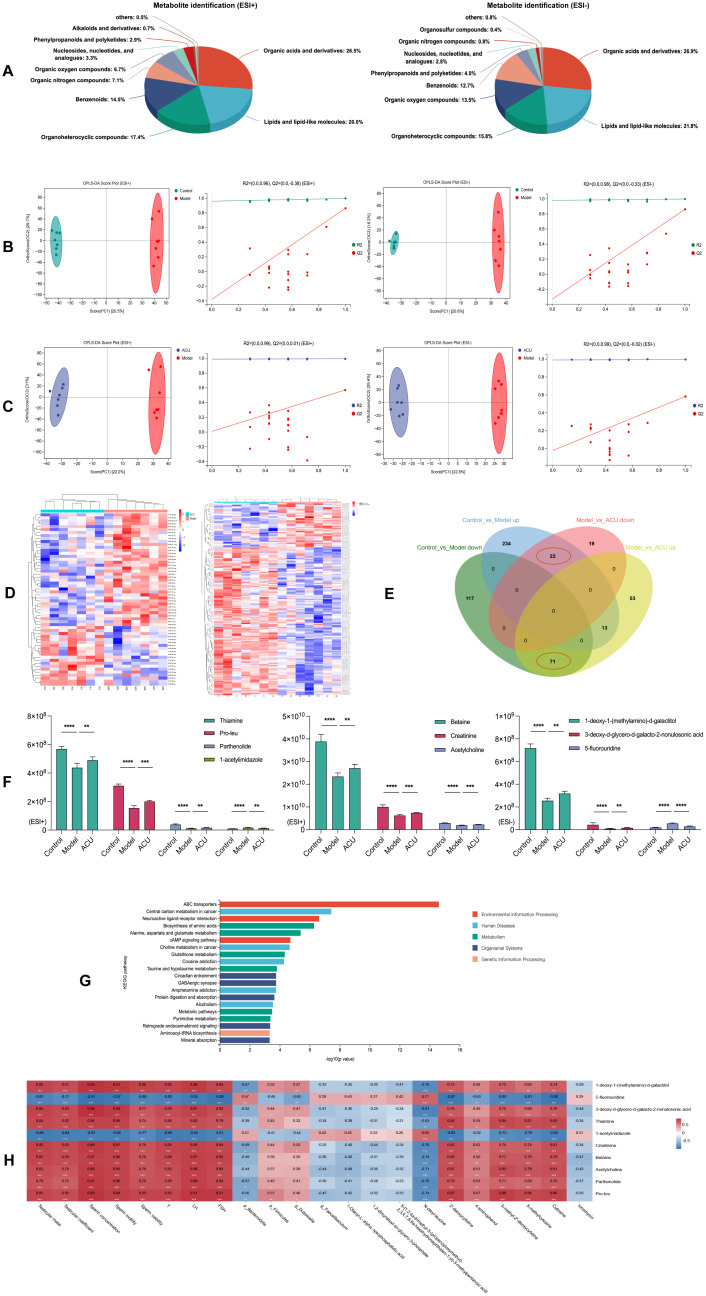
Acupuncture regulated testicular metabolic disorders in the asthenozoospermic mice. **(A)** Testicular metabolite identification and analysis in ESI+ and ESI- Modes. **(B)** OPLS-DA analysis and permutation tests between the control and model groups under ESI+ and ESI- modes. **(C)** OPLS-DA analysis and permutation tests between the model and ACU groups under ESI+ and ESI- modes. **(D)** Clustering analysis of testicular DEMs between groups under the combined ESI+ and ESI- modes. **(E)** Venn diagram of intersecting DEMs under the combined ESI+ and ESI- modes. **(F)** Comparison of expression levels of the top 10 DEMs by VIP values. **(G)** KEGG enrichment analysis. **(H)** Spearman correlation analysis. *P < 0.05, **P < 0.01, ***P < 0.001, ****P < 0.0001.

Harmful substances and immune cells may infiltrate the testes when the BTB is disrupted. This triggers local inflammation and immune responses that alter the metabolic environment within the testes. In addition, this disruption can impair the synthesis and secretion of testicular hormones. This will further interfere with normal metabolism (26). Therefore, we analyzed the metabolites in the testes of each group. All identified metabolites were categorized according to their chemical classifications. The results indicated that in both the ESI+ and ESI– modes, the metabolites predominantly belonged to four major categories: organic acids and derivatives, lipids and lipid-like molecules, organoheterocyclic compounds, and benzenoids, and they collectively accounted for approximately 80% of the total metabolites (**Figure 6A**). We then used the OPLS-DA model to detect the intergroup differences. The results showed an obvious separation between the control and model groups as well as between the model and ACU groups, and there existed a tight clustering of samples within each group (**Figures 6B, C**). The internal cross-validation of the model revealed that in the ESI+ mode, the R2Y and Q2Y values between the control and model groups were 0.998 and 0.863, respectively, and between the model and ACU groups they were 0.994 and 0.571, respectively. In the ESI– mode, the R2Y and Q2Y values between the control and model groups were 0.998 and 0.861, respectively, and between the model and ACU groups, they were 0.996 and 0.583, respectively. These findings suggested that the model had strong explanatory power but moderate predictability. We then conducted a permutation test to further validate the model reliability. All of the Q2 points were lower than the original Q2 point on the far right (**Figures 6B, C**), and most Q2 points intersected the vertical axis at values less than zero. These findings indicated that the results of this study were reliable and valid.

In the combined ESI+ and ESI– modes, 457 DEMs were identified between the control and model groups (269 upregulated and 188 downregulated), and 178 DEMs were identified between the model and ACU groups (137 upregulated and 41 downregulated) (**Supplementary Table 2**). The clustering heatmap revealed distinct differences between the control vs. model groups and the model vs. ACU groups (**Figure 6D**). These findings indicated that the results were reliable and suitable for further analysis. The Venn diagram (**Figure 6E**) showed that 22 DEMs were upregulated in the model group and downregulated after acupuncture treatment, while 71 DEMs were downregulated in the model group and upregulated after acupuncture treatment. In addition, we highlighted the top 10 DEMs with the highest VIP values (**Figure 6F**). The KEGG functional enrichment analysis of these 93 intersecting DEMs revealed that they were primarily associated with metabolic pathways such as ABC transporters, neuroactive ligand–receptor interaction, and the biosynthesis of amino acids (**Figure 6G**). A Spearman correlation analysis was conducted to investigate the relationships between the top 10 testicular DEMs that had high VIP scores and the key parameters of asthenozoospermia, the gut microbiota, and the serum DEMs (**Figure 6H**). 5-fluorouridine and 1-acetylimidazole were strongly negatively correlated with the sperm parameters and the serum hormone levels (*P* < 0.01). By contrast, the remaining testicular DEMs exhibited strong positive correlations with these parameters (*P* < 0.01). In addition, most testicular DEMs showed varying degrees of correlation with the serum DEMs (*P* < 0.01, *P* < 0.05).

### FMT improved reproductive function in the asthenozoospermic mice

**Figure 7:**
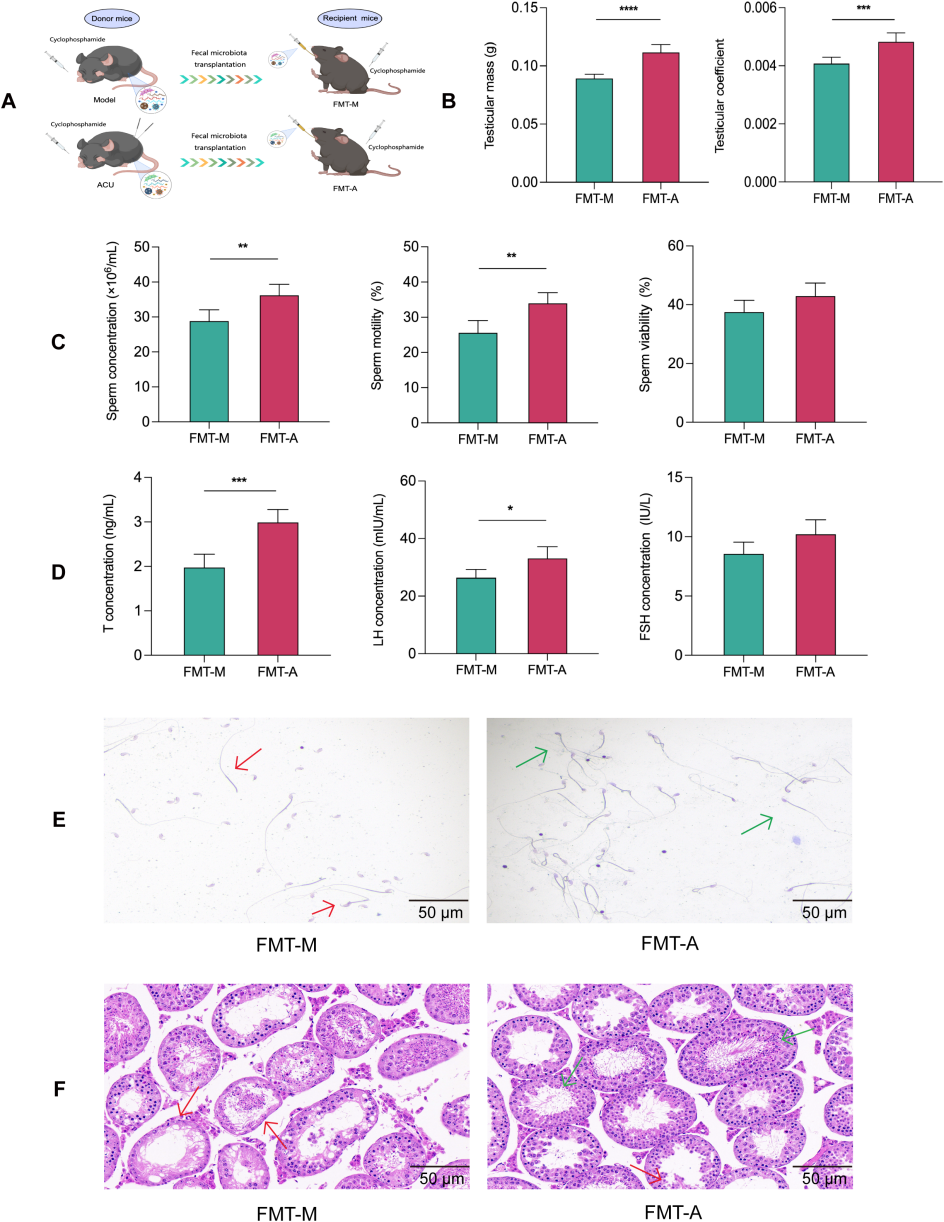
FMT improved reproductive function in the asthenozoospermic mice. **(A)** Scheme of FMT experiment. **(B)** Comparison of testicular mass and testicular coefficient of each group (n=6). **(C)** Comparison of sperm parameters of each group (n=3). **(D)** Comparison of serum T, FSH, and LH concentrations of each group (n=6). **(E)** Comparison of sperm pathological morphology of each group (n=3). **(F)** Comparison of testicular histopathological morphology of each group (n=6). In **(E)** red arrows indicate abnormal sperm morphology, such as headless sperm and sperm with abnormal tail bending or folding, while green arrows indicate normal sperm morphology. In **(F)** red arrows indicate seminiferous tubules with uneven wall thickness and dilated lumens, while green arrows indicate seminiferous tubules with improved structural integrity. Data are presented as mean ± SD. *P < 0.05, **P < 0.01, ***P < 0.001, ****P < 0.0001.

We conducted further verification using an FMT experiment to determine whether the reproductive protective effects of acupuncture on the asthenozoospermic mice were dependent on the gut microbiota (**Figure 7A**).Compared with the FMT-M group, the FMT-A group showed varying degrees of improvement in testicular weight, testicular index, sperm concentration, sperm motility, and serum levels of T and LH (P < 0.01, P < 0.05). These results were consistent with the results observed in acupuncture-treated mice (**Figures 7B–D**). In addition, the FMT-M group had a lower sperm count and abnormal morphology that included irregular head size and shape, abnormal tail bending or folding, and these could all potentially affect sperm motility and fertilization capability (27). The testes of the FMT-M group also showed significant morphological abnormalities and structural disorganization. By contrast, the sperm and testicular morphology and structures of the FMT-A group improved to varying degrees (**Figures 7E, F**). In conclusion, these results suggested that the reproductive protective effects of acupuncture can be achieved using FMT.

## Discussion

Human reproduction is directly related to sperm motility, and it is a crucial male fertility indicator. Normal forward motility is essential for sperm to travel from the vagina to the ampulla of the fallopian tube and successfully fertilize the egg (28). The WHO refers to reduced sperm motility as asthenozoospermia (2). Acupuncture is a significant component of complementary and alternative medicine and is a common non-pharmacological treatment method that can adjust the body’s endocrine balance and improve reproductive system function by stimulating specific acupoints. Research has indicated that acupuncture may enhance sperm motility and counts through various mechanisms that include the promotion of local blood circulation, the alleviation of oxidative stress, the modulation of immune defenses, and the correction of hormonal imbalances (10–12, 29, 30). In this study, we applied the “Zhibian (BL 54)-to-Shuidao (ST 28)” acupuncture technique to a cyclophosphamide-induced asthenozoospermia mice model. The findings demonstrated that acupuncture effectively corrected hormonal imbalances in the asthenozoospermic mice, improved testicular tissue pathological damage, and enhanced sperm quality.

Recent scientific research has increasingly highlighted the critical role of the gut microbiota in male reproductive health. Studies have shown that dysbiosis, such as a changed microbial diversity or an increase in harmful bacteria, may be associated with decreased sperm quality. The gut microbiota can directly impact sperm production and quality through several mechanisms that include hormone level regulation, oxidative stress reduction, and inflammatory response control. In addition, gut microorganisms can influence the overall physiological status through nutrient absorption and metabolism and indirectly affect male reproductive health (31, 32). Therefore, an analysis of the gut microbiota in mice from each group was conducted in this study. The microbial composition of the samples primarily included Bacteroidota, Firmicutes, Actinobacteriota, and Proteobacteria. This phylum-level composition and its relative abundance are basically consistent with the results of previous studies on the population with asthenozoospermia (33). The asthenozoospermic mice exhibited significantly reduced abundances of *p*_Bacteroidota and *g*_*Faecalibaculum* compared with the control group. The abundances of *p*_Firmicutes and *g*_*Dubosiella* were significantly increased compared with the control group. These differential microbial communities were correlated with indicators such as sperm concentration and motility, and acupuncture treatment reversed these changes. Bacteroidota is considered a potentially harmful phylum and has been shown in several studies to negatively correlate with sperm concentration and motility (34). Similarly, the Firmicutes phylum is closely related to reproductive health (35), and the genera *Dubosiella* and *Faecalibaculum* are important members of the Firmicutes phylum. They have been found to be abnormally expressed in male testicular dysfunction or female ovarian dysfunction (36–39). Furthermore, the metabolic pathway predictions for the intersecting microbial species revealed that they were primarily related to amino acid biosynthesis and nucleoside and nucleotide biosynthesis. Amino acids are fundamental components of proteins and are involved in structural and functional protein synthesis within sperm cells, supporting sperm morphology and motility (40). In addition, amino acids play critical roles in energy metabolism and antioxidant defense, and they protect sperm from oxidative stress (41). Nucleotides ensure the accurate transmission of genetic information in sperm and are also involved in various cellular activities such as energy metabolism and signal transduction (42). This ensures that sperm can obtain sufficient energy to perform the vigorous flagellar movements necessary for motility and ultimately fertilization. Therefore, acupuncture may improve sperm motility in asthenozoospermic mice by regulating these various pathways.

ZO-1, Claudin-1, and Occludin are crucial components of tight junctions in the intestinal barrier (43). ZO-1 maintains tight junction integrity by linking tight junction proteins to the cytoskeleton. Claudin-1 regulates the permeability of intercellular spaces to ensure the barrier function. Occludin stabilizes tight junction structures while participating in cell permeability and signal transduction regulation. These proteins work together to ensure intestinal barrier integrity and function to prevent pathogens and harmful substances from passing through epithelial cells into the body, thus maintaining intestinal health and homeostasis. We observed in this study that cyclophosphamide-induced mice exhibited the downregulation of ZO-1, Claudin-1, and Occludin expressions, indicating increased intestinal permeability and suggesting that harmful substances may more readily enter the bloodstream, thereby affecting serum metabolism. Consequently, the serum metabolomics of the mice from each group were then further analyzed. The results demonstrated that compared with the control group, the expression levels of DEMs, such as cytosine, 2’-deoxycytidine, 5-methylcytosine, 5-methyl-2’-deoxycytidine, and 4-aminophenol, were decreased in the serum of asthenozoospermia mice, while the levels of 1,2-dimyristoyl-sn-glycero-3-phosphate, 5-[1,2,4a-trimethyl-5- (propanoyloxymethyl)-2,3,4,7,8,8a-hexahydronaphthalen-1-yl]-3-methylpentanoic acid, 1-Oleoyl-L-.alpha.-lysophosphatidic acid, N-oleyl-leucine, and ionomycin were increased. Cytosine is a fundamental DNA component that ensures accurate replication and transmission of sperm genetic information (44). 5-Methylcytosine is a methylated form of cytosine that regulates gene expression, thereby affecting sperm fertilization capability and subsequent embryonic development (45). 5-Methyl-2’-deoxycytidine, the deoxynucleotide form of 5-methylcytosine, is crucial to maintain the epigenetic marks and gene expression regulation in sperm DNA (46). In addition, 4-aminophenol may induce oxidative stress, and this can lead to sperm DNA damage, genomic instability, and compromised sperm quality (47). Hence, when the metabolism or modification of cytosine and its related compounds is disrupted or deficient, this may lead to sperm DNA damage or abnormal gene expression. This disruption will then reduce sperm quality and impair male fertility. Moreover, as phospholipid compounds or fatty acid derivatives, elevated levels of 5-[1,2,4a-trimethyl-5-(propanoyloxymethyl)-2,3,4,7,8,8a- hexahydronaphthalen-1-yl]-3-methylpentanoic acid, 1-Oleoyl-L-α-lysophosphatidic acid, 1,2-dimyristoyl-sn-glycero-3-phosphate, and N-oleyl-leucine may alter the lipid composition of the sperm membrane, compromise membrane stability, reduce sperm motility, and impair the normal sperm membrane function (48, 49). Ionomycin, a calcium ionophore, can lead to abnormal intracellular calcium levels when present in excess. This will disrupt sperm motility (50). Furthermore, the KEGG analysis revealed that these DEMs were primarily associated with metabolic pathways such as pyrimidine metabolism and protein digestion and absorption. Proper pyrimidine metabolism may exert antioxidant effects that are critical to maintain sperm DNA stability and to enhance sperm motility. In addition, efficient protein digestion and absorption ensure the availability of sufficient amino acids to support sperm formation and maturation (51, 52). Consequently, the aberrant states of these metabolites are often closely linked to declines in sperm quality. Following the acupuncture treatment, not only were the alterations in these metabolites reversed, but the expression levels of ZO-1, Claudin-1, and Occludin in the colonic tissues also increased. This result suggested that acupuncture may ameliorate serum metabolic disturbances by improving the gut barrier function.

The BTB is a critical structure within mammalian testes. It is primarily composed of tight junctions, gap junctions, and adherens junctions. It separates the basal compartment from the luminal compartment of the seminiferous tubules, creating two distinct microenvironments: the basal area and the luminal area. The BTB helps maintain appropriate ion concentrations, pH, and nutrient levels within the seminiferous tubules, and it provides a stable spermatogenesis environment. By isolating germ cells from harmful substances in the blood and lymph, the barrier prevents immune system attacks on germ cells. This ensures uninterrupted sperm production and maturation. The BTB also regulates molecular exchanges between the tubule interior and exterior, and it allows only specific substances to pass through it (53, 54). Occludin, Connexin-43, and N-cadherin are key components of this barrier. Occludin is a tight junction protein that maintains the seal between cells and prevents the passage of large molecules. Connexin-43 is a gap junction protein that facilitates the transfer of small molecules and ions between supporting cells, and this ensures synchronized intercellular signaling. N-cadherin is an adherens junction protein that maintains cellular structural stability through calcium-dependent adhesion (55). These molecules collectively ensure BTB integrity and functionality, which is crucial for sperm production and male reproductive health. We observed abnormal pathological structures in the testicular tissues of the model group mice in this study, and this was accompanied by varying degrees of reduced expressions of Occludin, Connexin-43, and N-cadherin. These results suggested increased BTB permeability. Metabolites, toxins, and inflammatory factors from the serum may more readily enter the testes due to this increased permeability. This will negatively affect testicular metabolomics. We further analyzed the testicular metabolomics of mice from each group. The asthenozoospermic mice testes had increased expression levels of 5-fluorouridine and 1-acetylimidazole among the top 10 DEMs of the VIP compared with the control group. The expression levels of betaine, creatinine, acetylcholine, thiamine, and parthenolide were decreased compared with the control. 5-Fluorouridine is a commonly used anticancer compound that may cause sperm DNA damage by interfering with RNA and DNA synthesis, thereby affecting sperm quality (56). Betaine can enhance sperm vitality by regulating oxidative stress (57), while creatinine is used as an important predictor of sperm quantity and quality by reflecting overall metabolic health (58). Acetylcholine is a neurotransmitter that has been shown to regulate sperm motility and fertilization capacity (59). Thiamine participates in energy metabolism and ensures that sperm have sufficient energy for motility (60). Parthenolide has anti-inflammatory and antioxidative stress properties that may protect sperm health (61). Furthermore, the KEGG analysis revealed that the intersecting DEMs were primarily associated with metabolic pathways such as ABC transporters and neuroactive ligand–receptor interactions. Studies have shown that ABC transporters can regulate the transmembrane substance transport to maintain the intracellular and extracellular environmental balance of sperm cells (62). The neuroactive ligand–receptor interaction can influence sperm signaling and motility behavior through interactions between neuroactive ligands and receptors (63). The proper functioning of these pathways is crucial for sperm health and fertility maintenance. After the acupuncture treatment, the Occludin, Connexin-43, and N-cadherin expression levels in the testes of the mice increased, and the aforementioned metabolic changes were reversed. Therefore, acupuncture may correct testicular metabolic disorders by improving the BTB.

**Figure 8:**
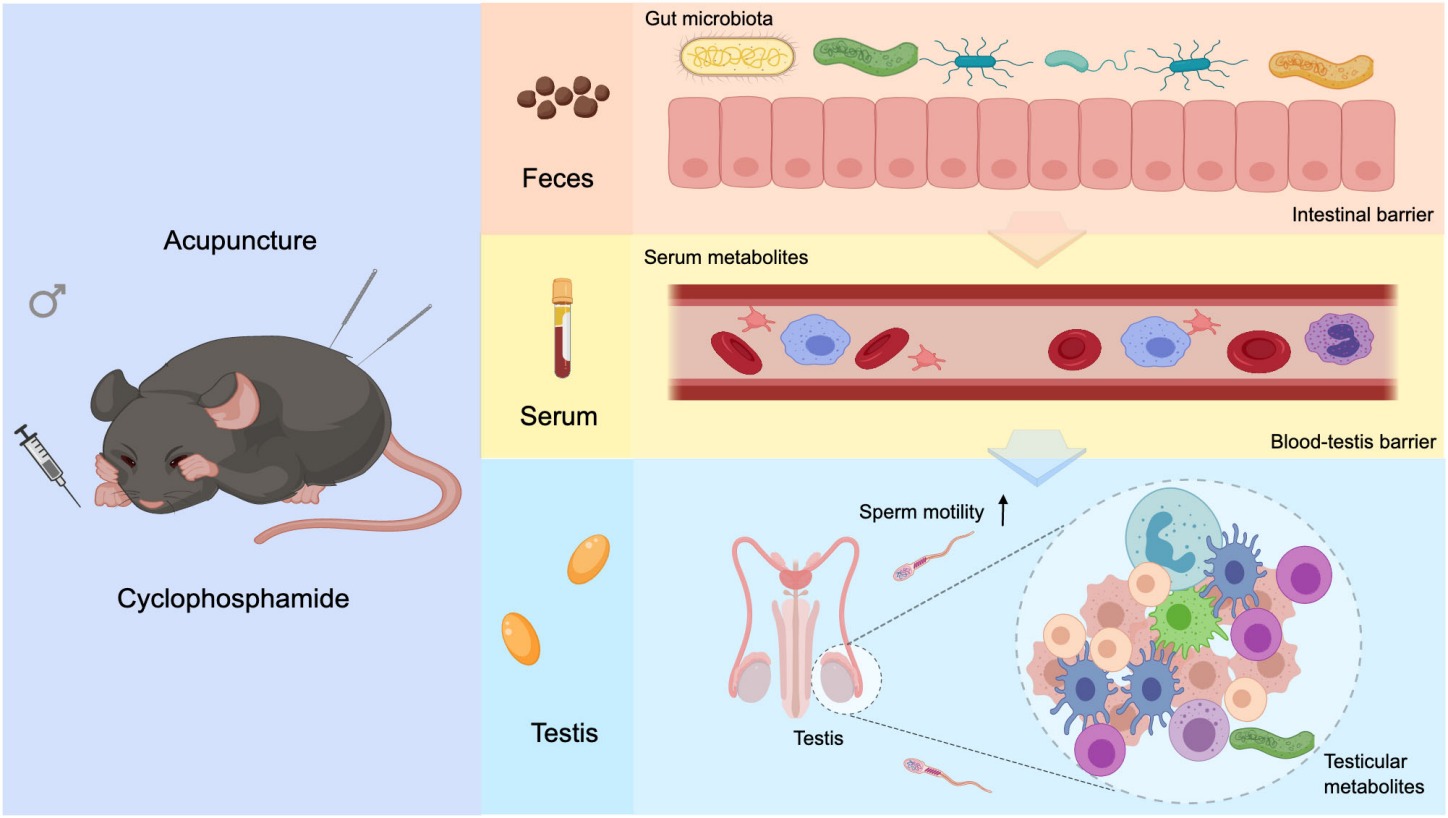
Graphical abstract. Acupuncture modulates gut microbiota composition and structure in asthenozoospermic mice, enhancing intestinal barrier integrity and correcting serum metabolic imbalances. This leads to restoration of BTB function and regulation of testicular metabolism, ultimately improving sperm motility.

The “gut–testis axis” is believed to mediate the connection between the gut microbiota and the reproductive system. Gut microbiota dysbiosis can lead to enhanced systemic inflammation, metabolic disorders, and hormonal imbalances, all of which may impair testicular function and consequently reduce sperm motility and quantity (17, 18, 64). Research by Li et al. indicated that chronic alcohol consumption disrupts the gut microbiota, alters the gut metabolites, and transmits these changes to the testis via the bloodstream. This results in testicular dysfunction (65). FMT is a technique that restores the recipient’s gut microbiota balance by transferring microbiota from healthy individuals, and it has gained significant attention in reproductive medicine. Studies have shown that FMT can restore diversity and stability to the recipient’s gut microbiota, improve the gut barrier function, and reduce toxin absorption. FMT may enhance the systemic metabolic environment and immune status and provide a healthier physiological sperm production environment (66, 67). We found that acupuncture-treated gut microbiota transplantation also caused varying degrees of improvement in the reproductive function of asthenozoospermic mice. Although it suggests that the restoration of gut microbiota may be related to improvements in sperm quality, FMT as a potential treatment strategy for male infertility still requires more direct evidence. In conclusion, the “gut–testis axis” is a complex network of interactions, and this study provides only preliminary evidence that acupuncture could improve asthenozoospermia through this axis (**Figure 8**). Further research is required to elucidate the unique roles of different gut microbiota in this process. Such research will provide a deeper understanding of this axis’s role in male reproductive health.

## Conclusion

Our findings indicated that acupuncture improved intestinal barrier damage by regulating the gut microbiota structure and function. These improvements modulated serum metabolic disorders, alleviated blood–testis barrier damage, and balanced testicular metabolic disorders. These ultimately enhanced sperm quality. This study provides theoretical support and new insights for acupuncture as a treatment for asthenozoospermia through the “gut–testis axis.”

## Data Availability

The datasets presented in this study can be found in online repositories. The names of the repository/repositories and accession number(s) can be found in the article/**Supplementary Material**.

## Supplementary material

Supplementary Table 1 Serum DEMs screening results.

Supplementary Table 2 Testicular DEMs screening results.

Supplementary Table 3 Gels and Blots image.
