# Supplementary figures and images for: Acupuncture mediates the “gut–testis axis” to improve asthenozoospermia
Source: Front Endocrinol (Lausanne). 2025 Jan 27;16:1514010. doi: 10.3389/fendo.2025.1514010 (PMC11827431; doi:10.3389/fendo.2025.1514010)

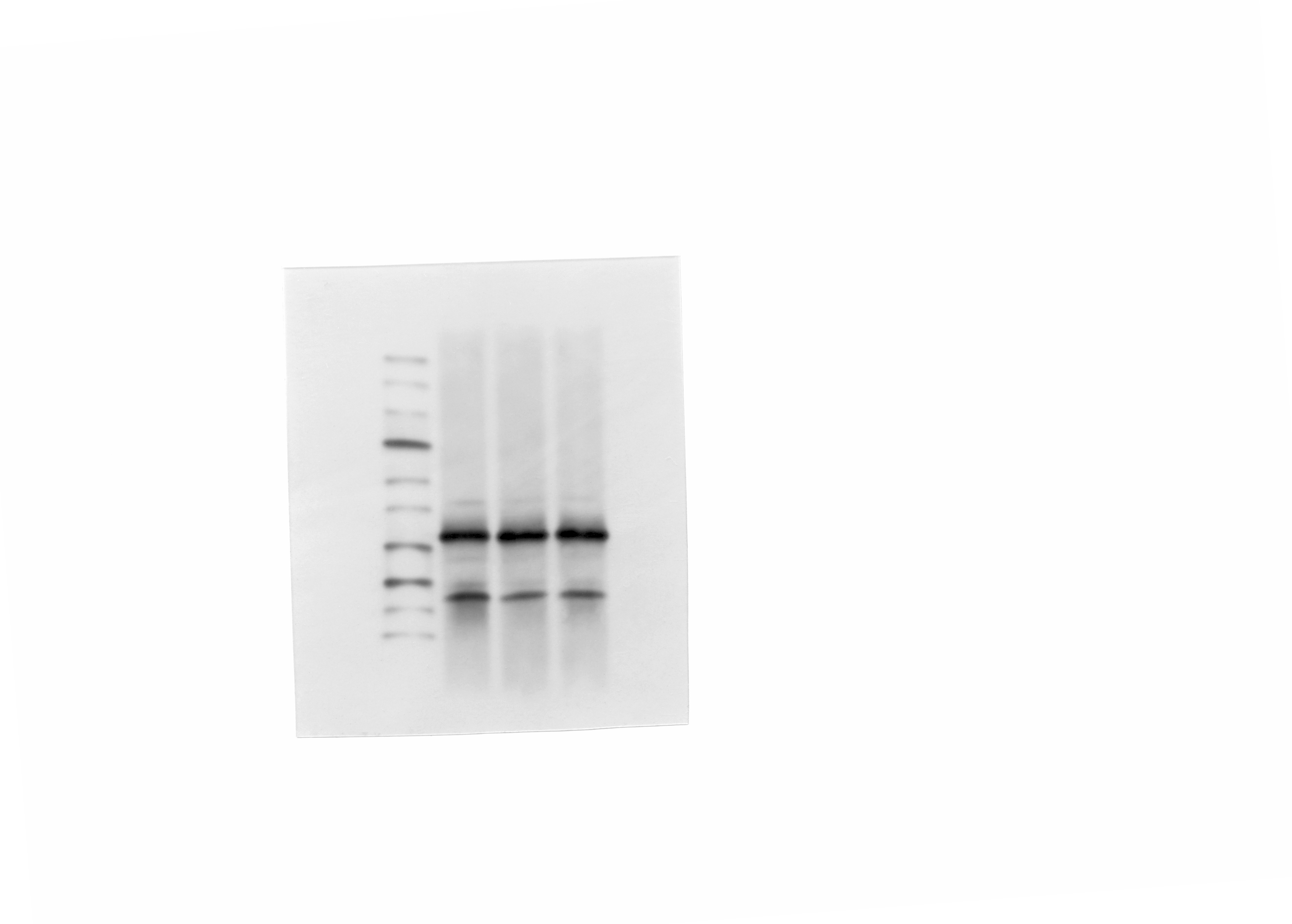

Supplement: Supplementary Table 3 — Gels and Blots image. [file DataSheet3.zip › Supplementary3 Gels and Blots image/Fig3B.Claudin-1∩╝ê2∩╝ë.tif]

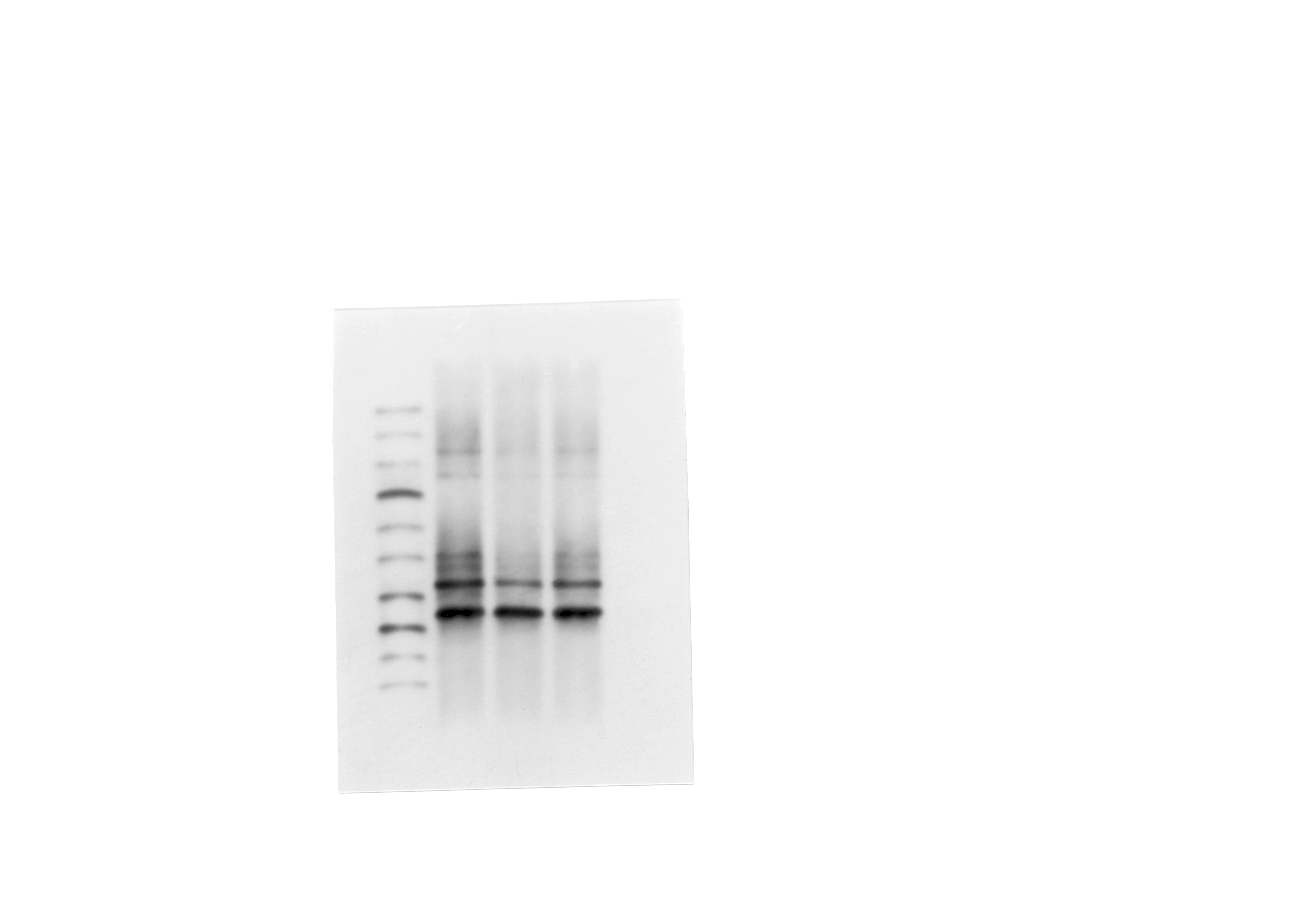

Supplement: Supplementary Table 3 — Gels and Blots image. [file DataSheet3.zip › Supplementary3 Gels and Blots image/Fig5A.Connexin-43∩╝ê2∩╝ë.tif]

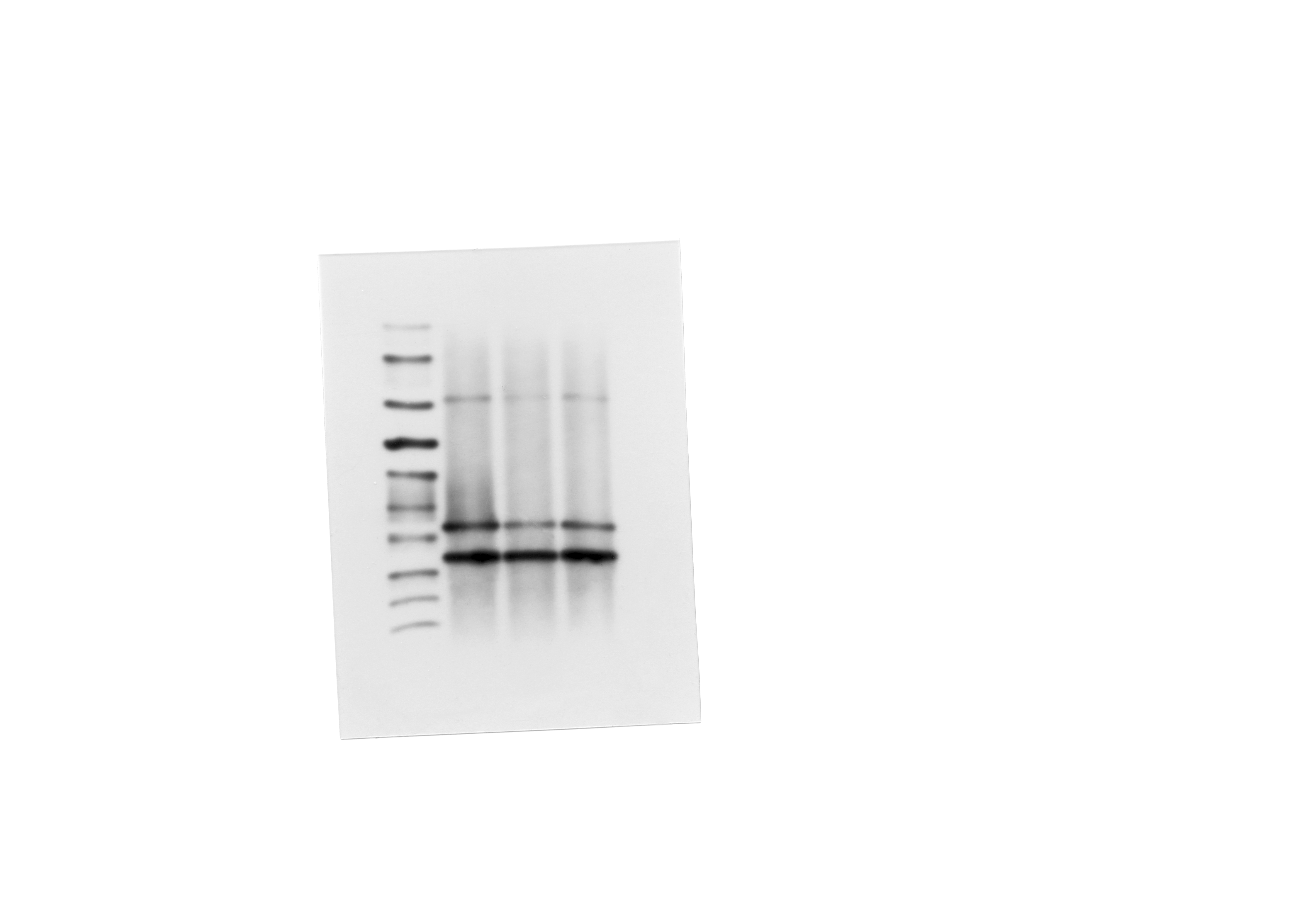

Supplement: Supplementary Table 3 — Gels and Blots image. [file DataSheet3.zip › Supplementary3 Gels and Blots image/Fig5A.Connexin-43∩╝ê3∩╝ë.tif]

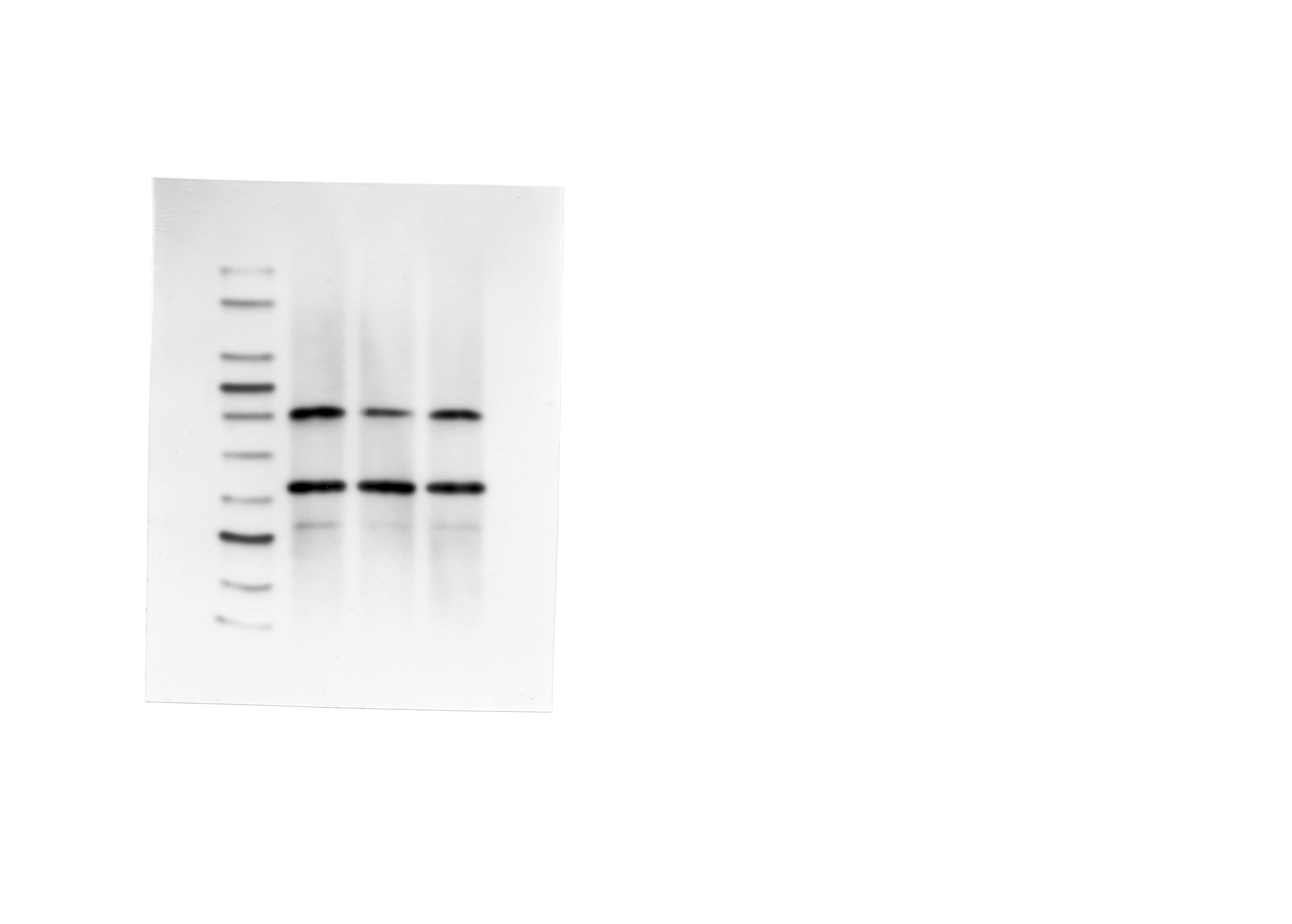

Supplement: Supplementary Table 3 — Gels and Blots image. [file DataSheet3.zip › Supplementary3 Gels and Blots image/Fig5A.Occludin∩╝ê1∩╝ë.tif]

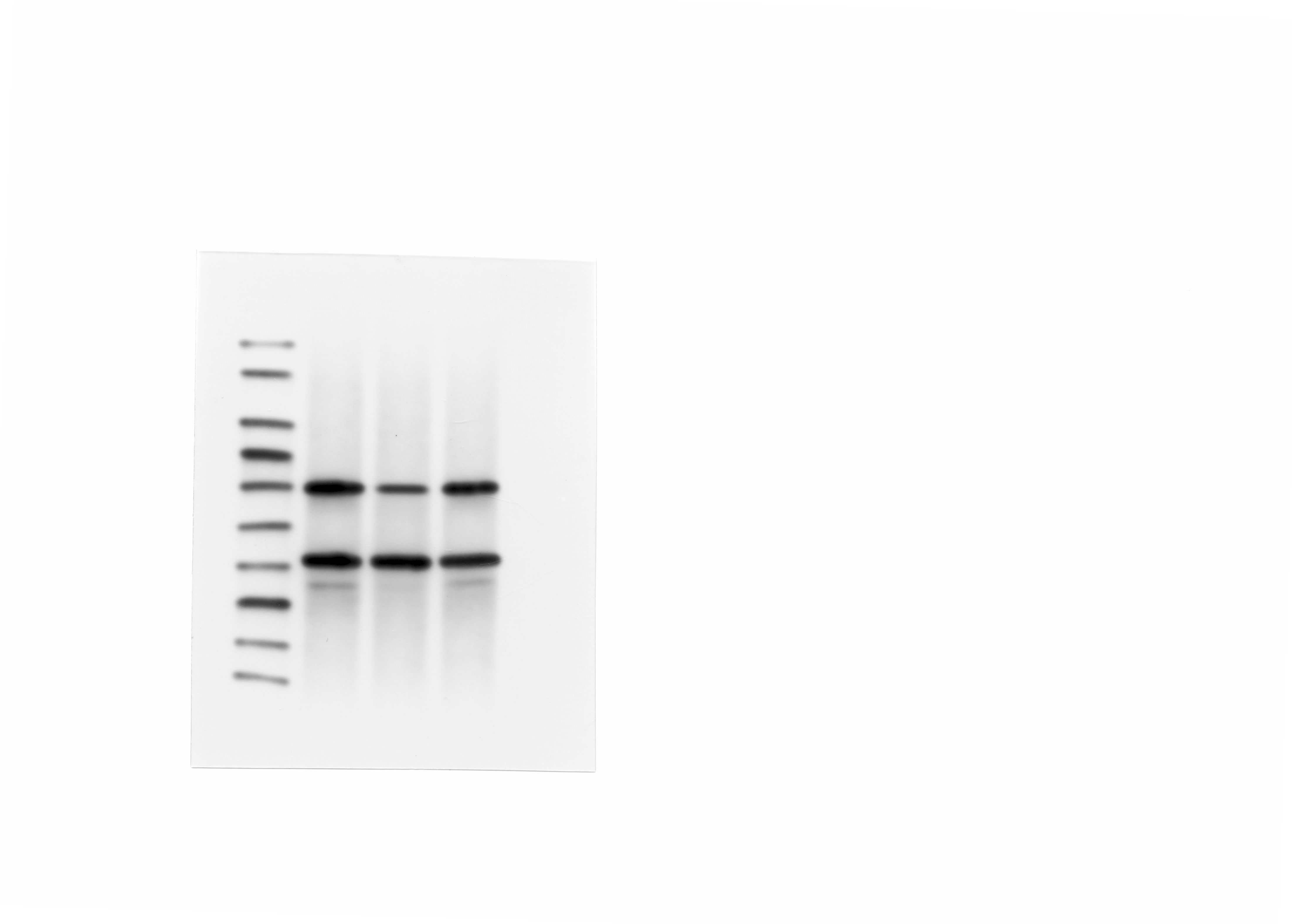

Supplement: Supplementary Table 3 — Gels and Blots image. [file DataSheet3.zip › Supplementary3 Gels and Blots image/Fig3B.Occludin∩╝ê1∩╝ë.tif]

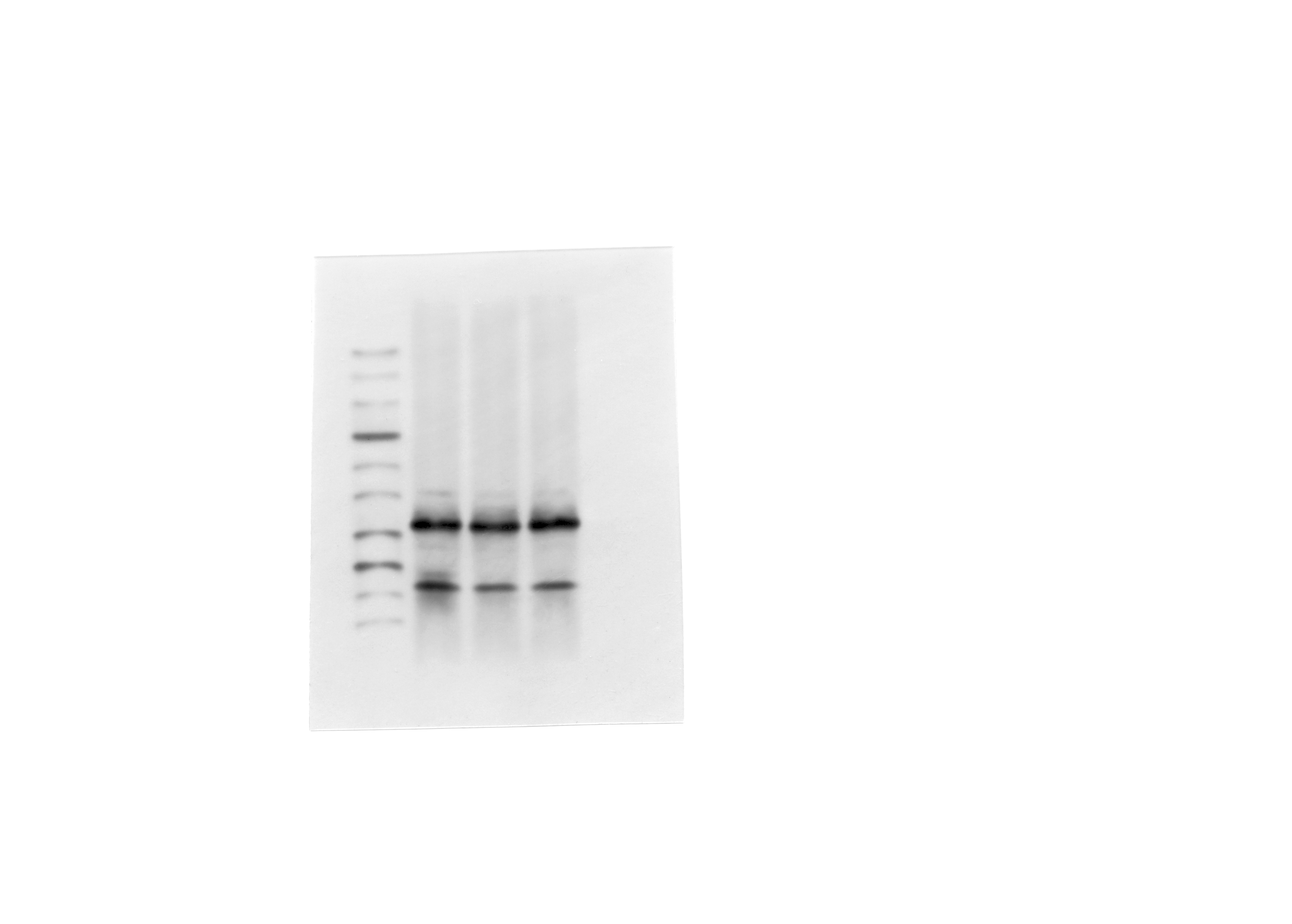

Supplement: Supplementary Table 3 — Gels and Blots image. [file DataSheet3.zip › Supplementary3 Gels and Blots image/Fig3B.Claudin-1∩╝ê3∩╝ë.tif]

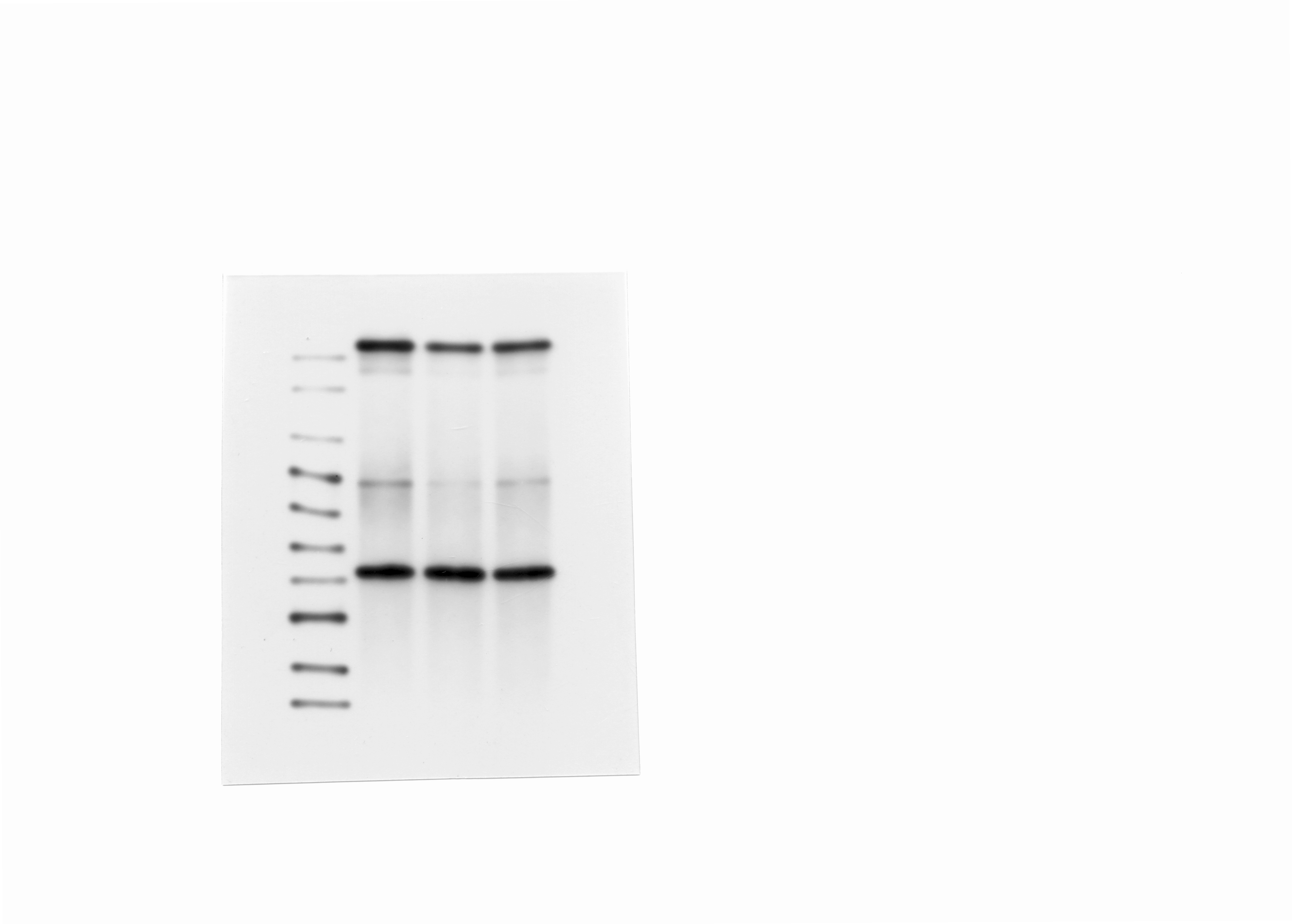

Supplement: Supplementary Table 3 — Gels and Blots image. [file DataSheet3.zip › Supplementary3 Gels and Blots image/Fig3B.ZO-1∩╝ê1∩╝ë.tif]

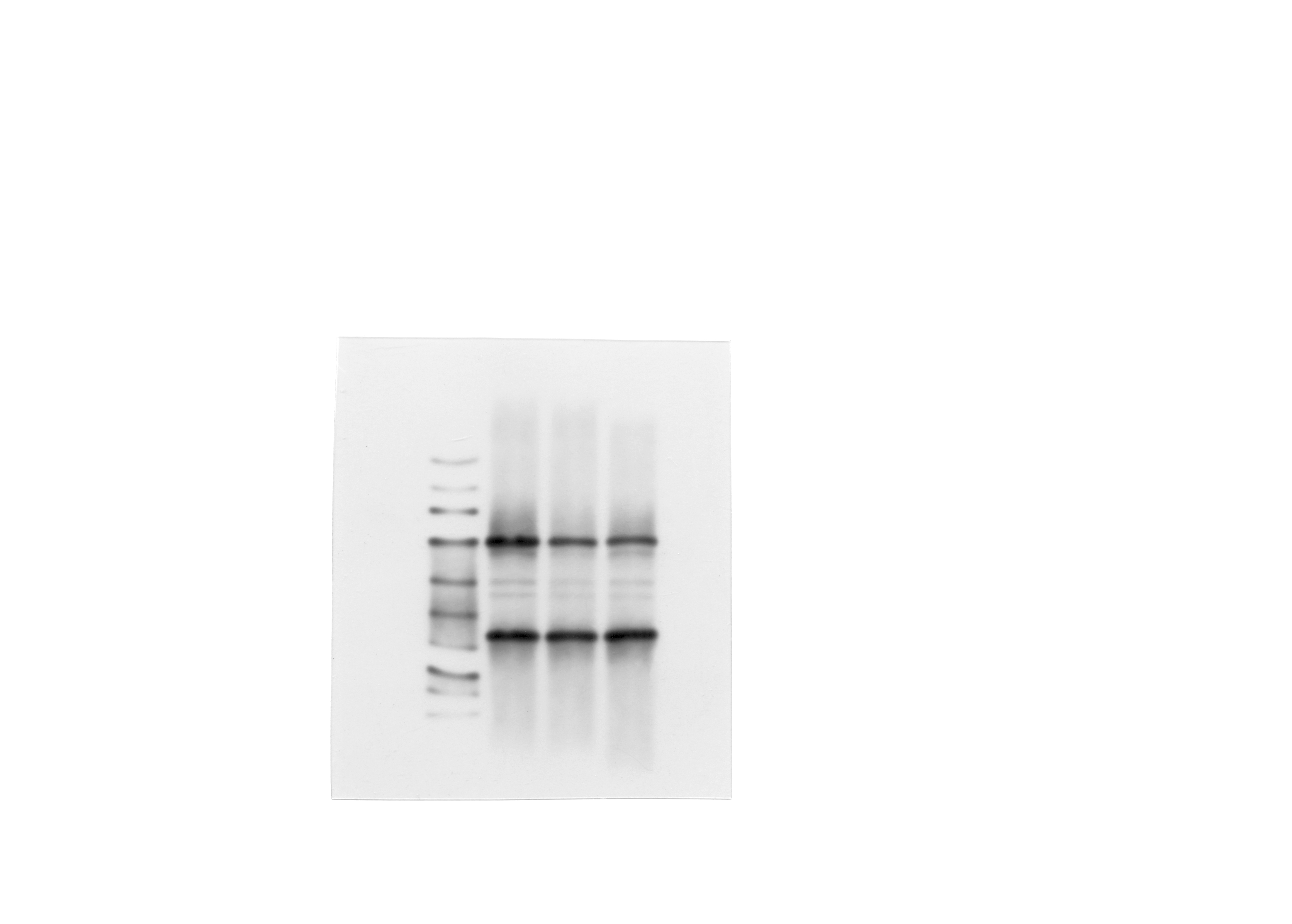

Supplement: Supplementary Table 3 — Gels and Blots image. [file DataSheet3.zip › Supplementary3 Gels and Blots image/Fig5A.N-cadherin∩╝ê2∩╝ë.tif]

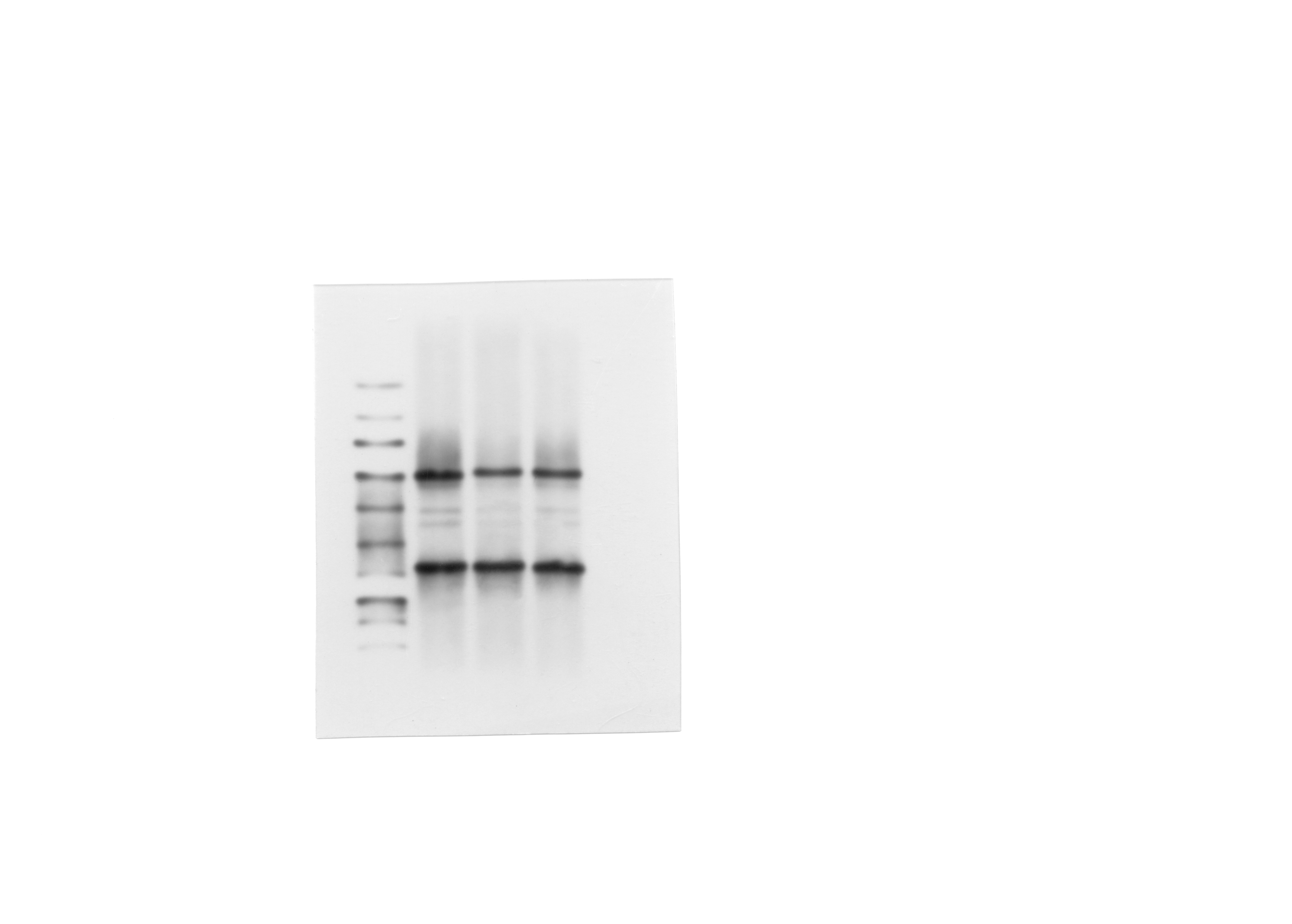

Supplement: Supplementary Table 3 — Gels and Blots image. [file DataSheet3.zip › Supplementary3 Gels and Blots image/Fig5A.N-cadherin∩╝ê3∩╝ë.tif]

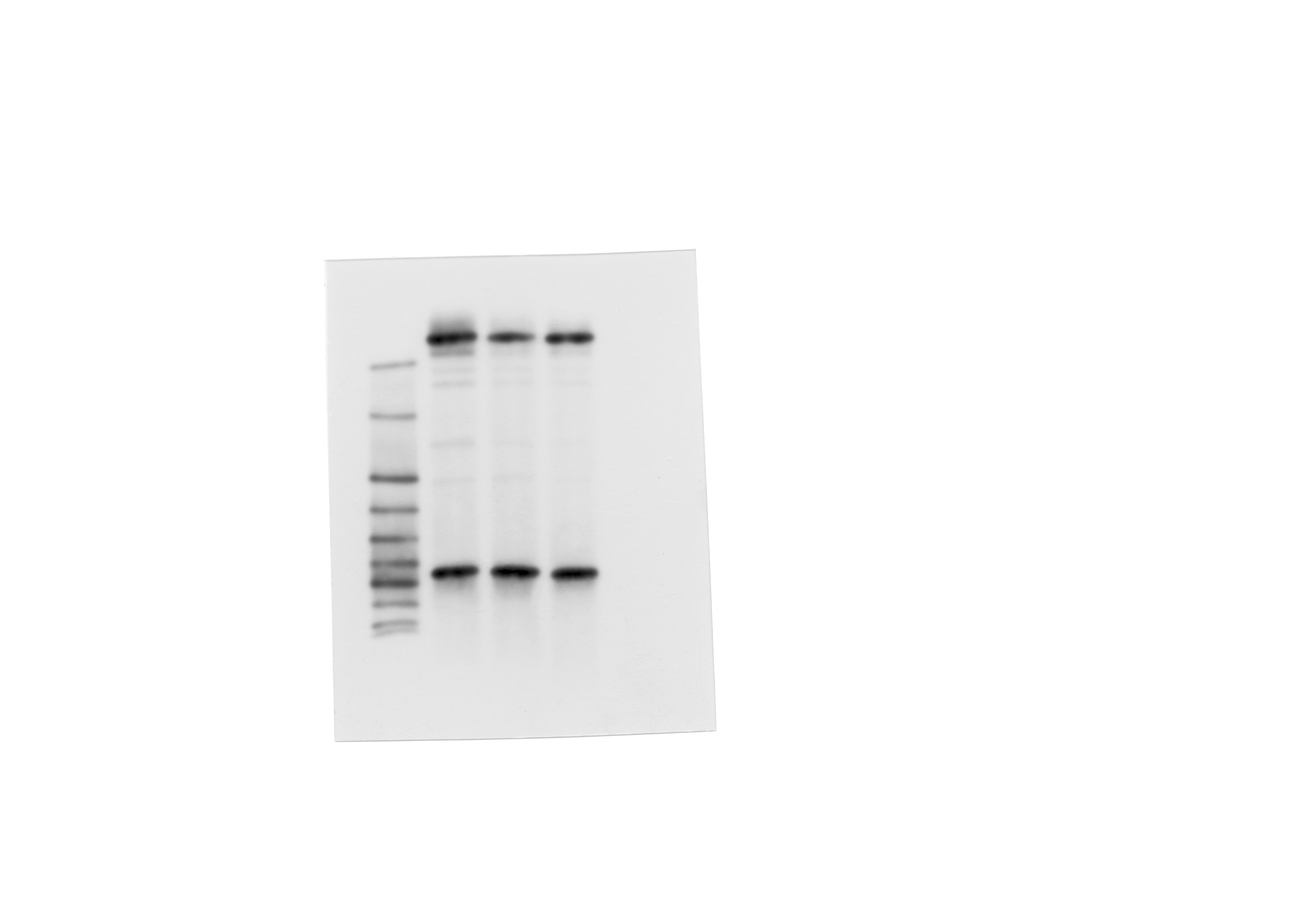

Supplement: Supplementary Table 3 — Gels and Blots image. [file DataSheet3.zip › Supplementary3 Gels and Blots image/Fig3B.ZO-1∩╝ê3∩╝ë.tif]

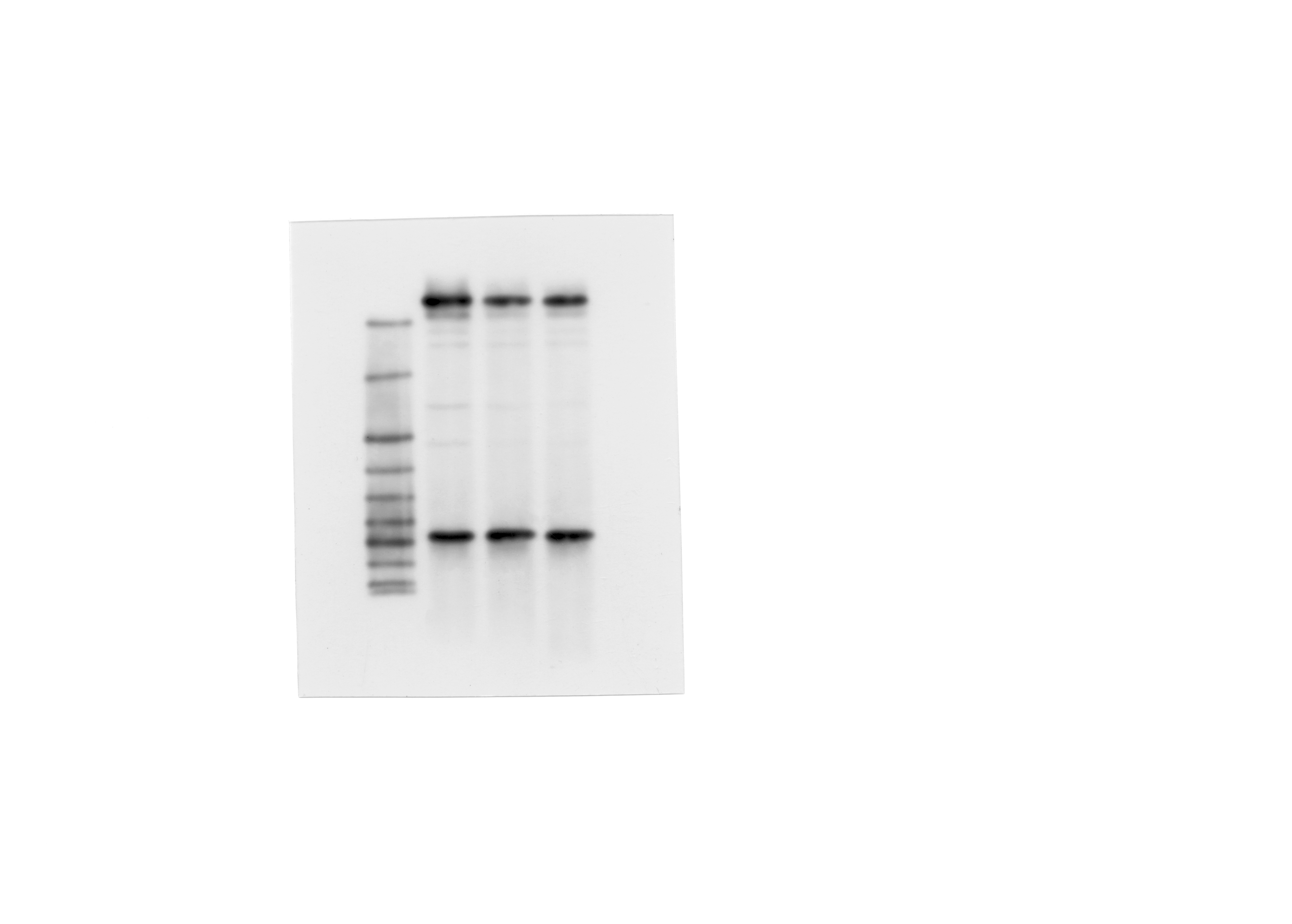

Supplement: Supplementary Table 3 — Gels and Blots image. [file DataSheet3.zip › Supplementary3 Gels and Blots image/Fig3B.ZO-1∩╝ê2∩╝ë.tif]

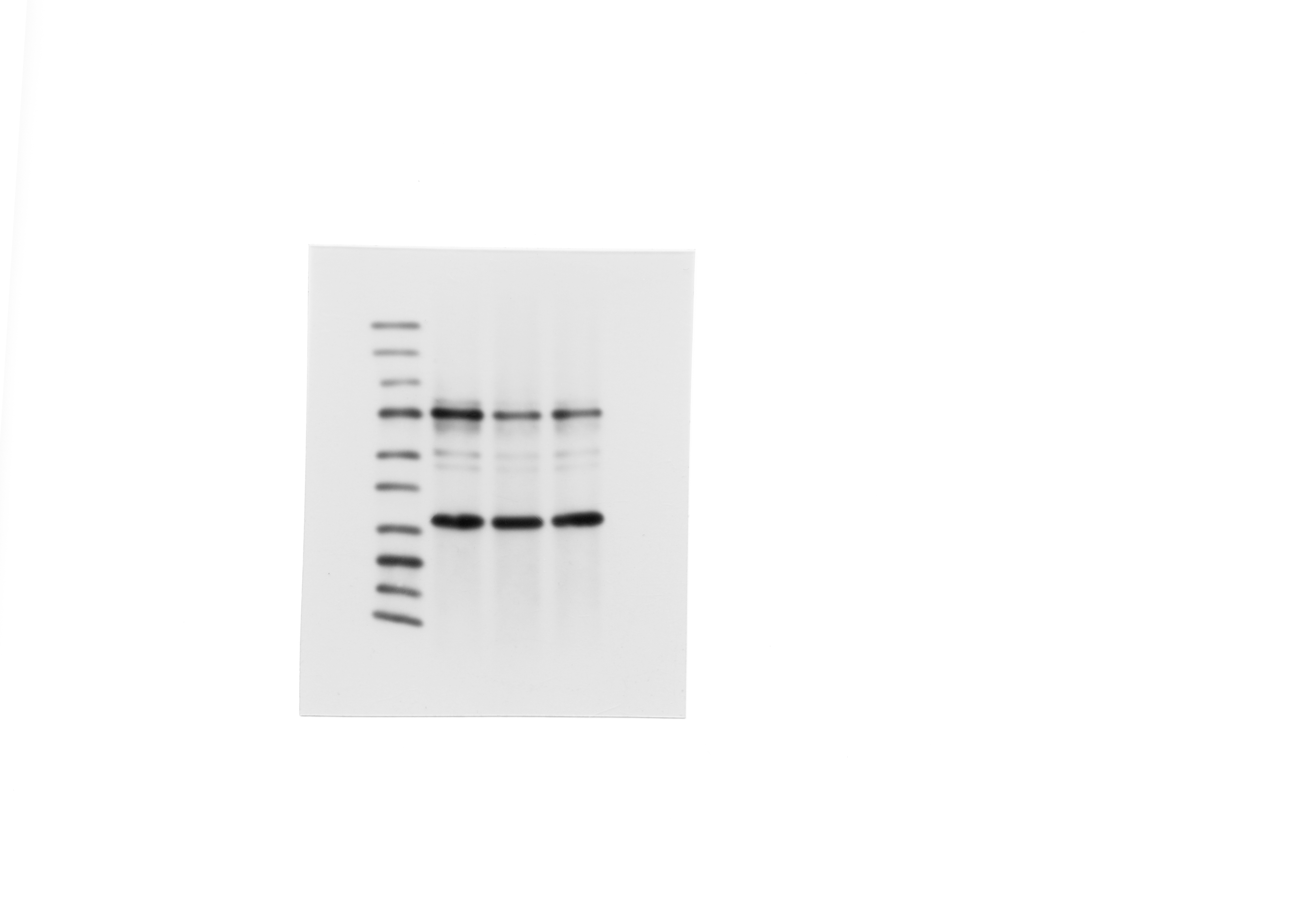

Supplement: Supplementary Table 3 — Gels and Blots image. [file DataSheet3.zip › Supplementary3 Gels and Blots image/Fig5A.N-cadherin∩╝ê1∩╝ë.tif]

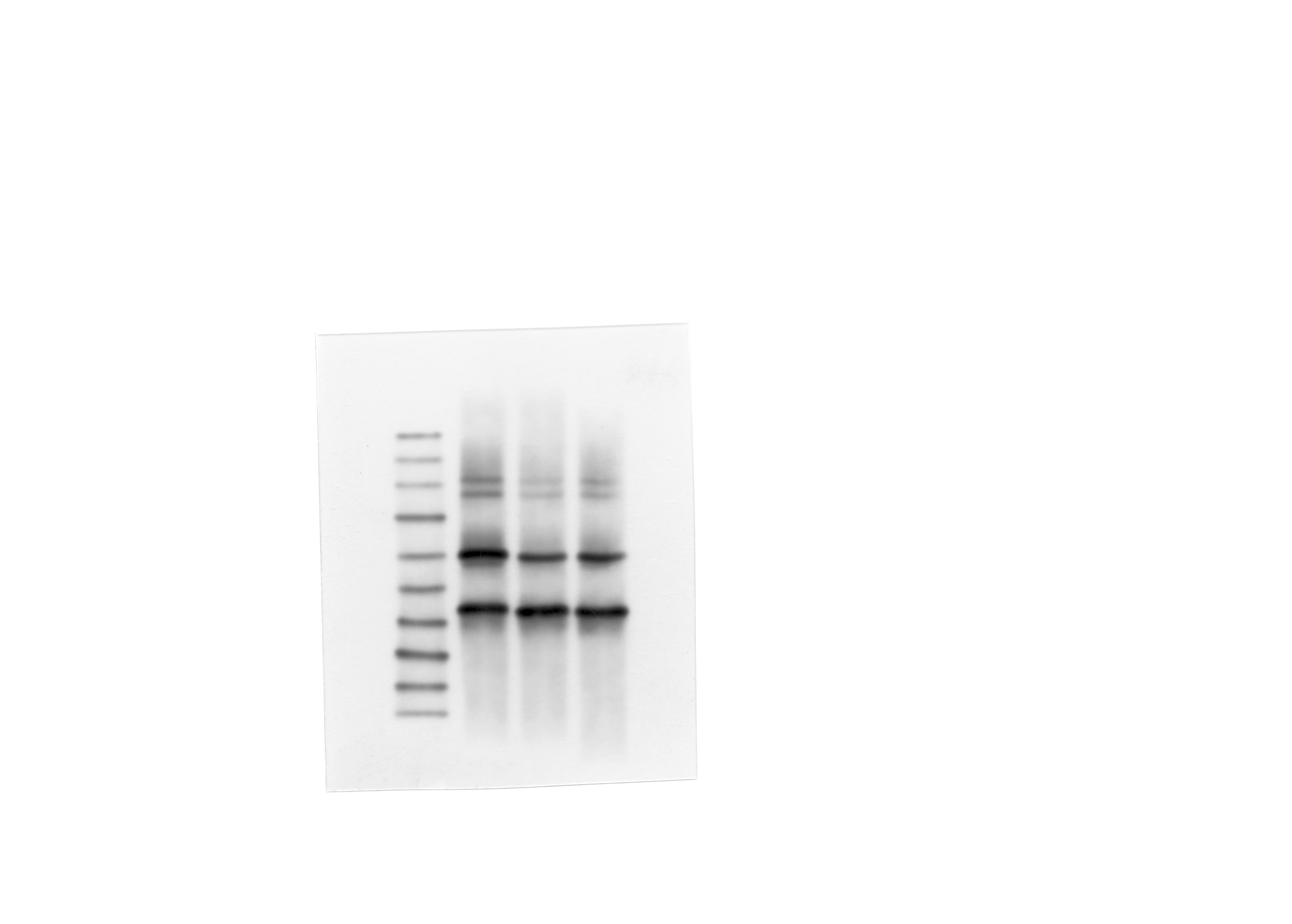

Supplement: Supplementary Table 3 — Gels and Blots image. [file DataSheet3.zip › Supplementary3 Gels and Blots image/Fig5A.Occludin∩╝ê2∩╝ë.tif]

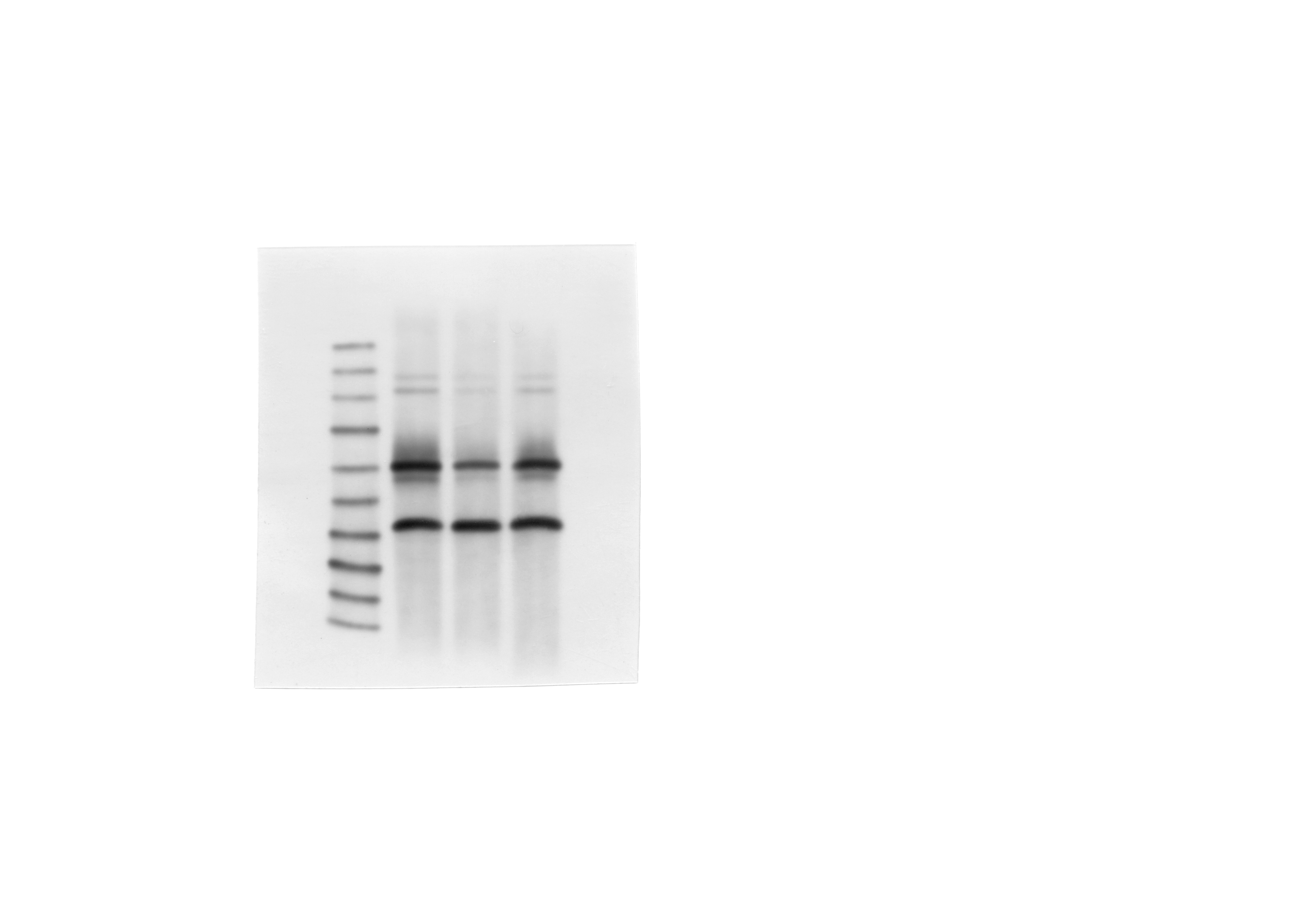

Supplement: Supplementary Table 3 — Gels and Blots image. [file DataSheet3.zip › Supplementary3 Gels and Blots image/Fig3B.Occludin∩╝ê2∩╝ë.tif]

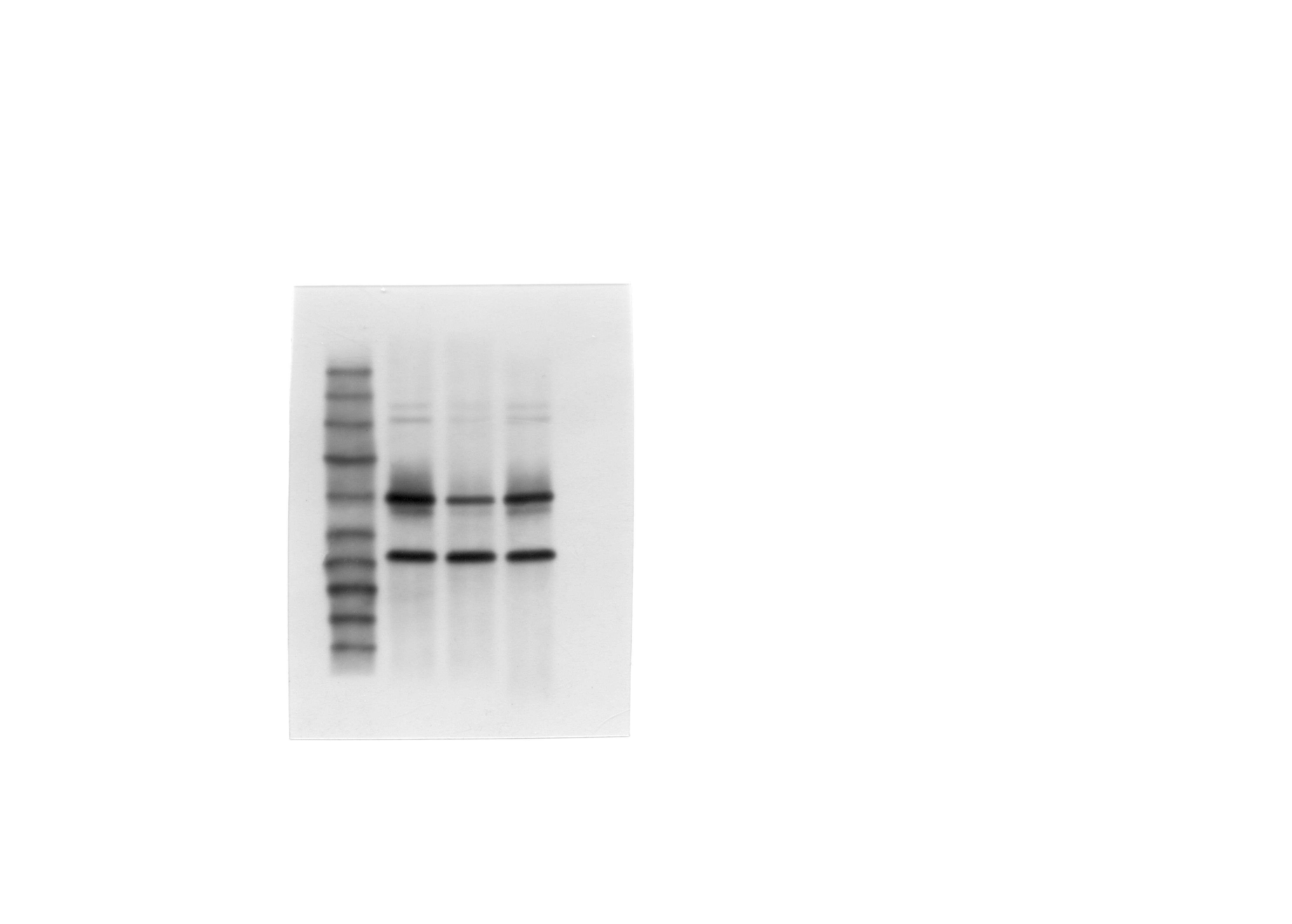

Supplement: Supplementary Table 3 — Gels and Blots image. [file DataSheet3.zip › Supplementary3 Gels and Blots image/Fig3B.Occludin∩╝ê3∩╝ë.tif]

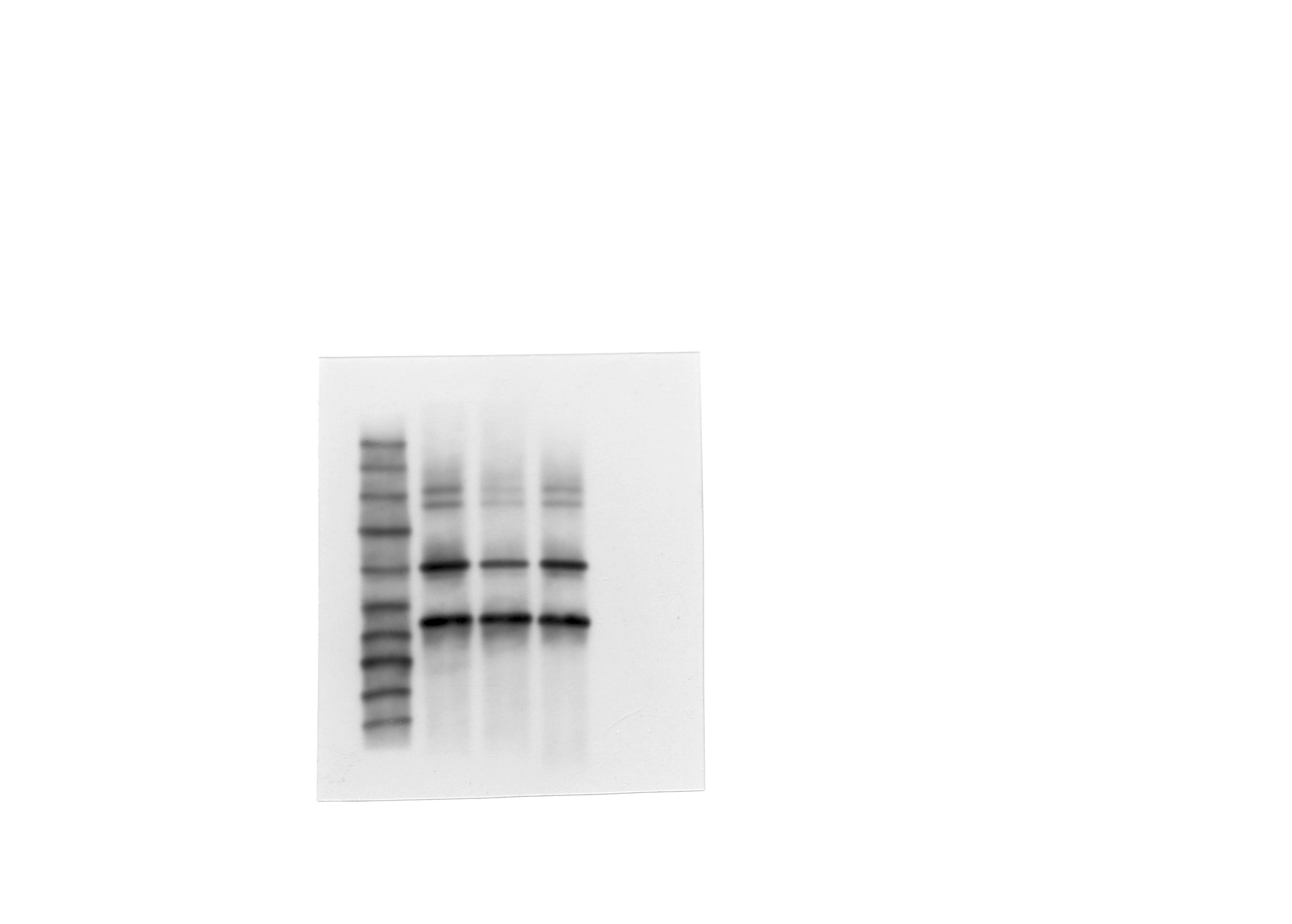

Supplement: Supplementary Table 3 — Gels and Blots image. [file DataSheet3.zip › Supplementary3 Gels and Blots image/Fig5A.Occludin∩╝ê3∩╝ë.tif]

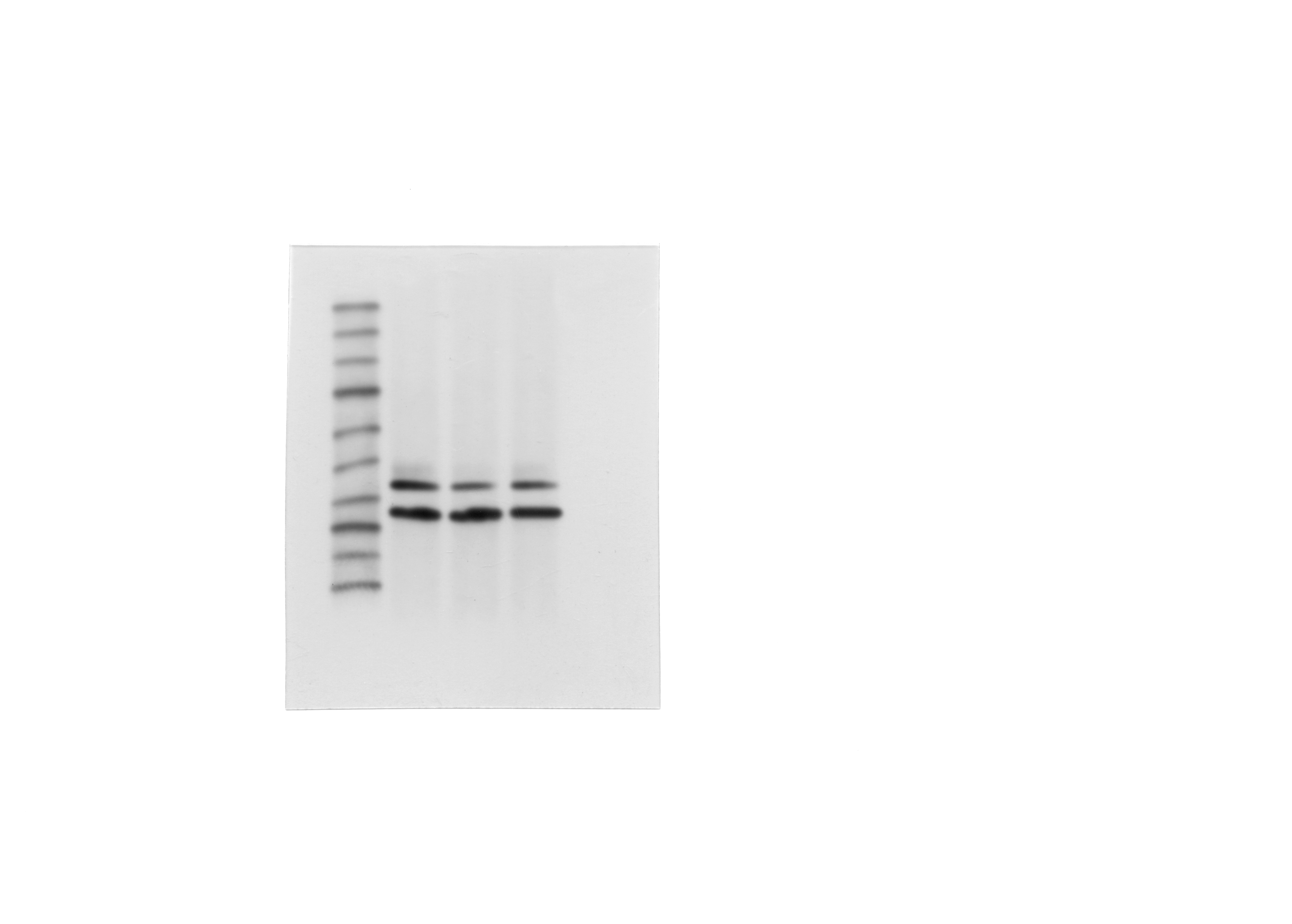

Supplement: Supplementary Table 3 — Gels and Blots image. [file DataSheet3.zip › Supplementary3 Gels and Blots image/Fig5A.Connexin-43∩╝ê1∩╝ë.tif]

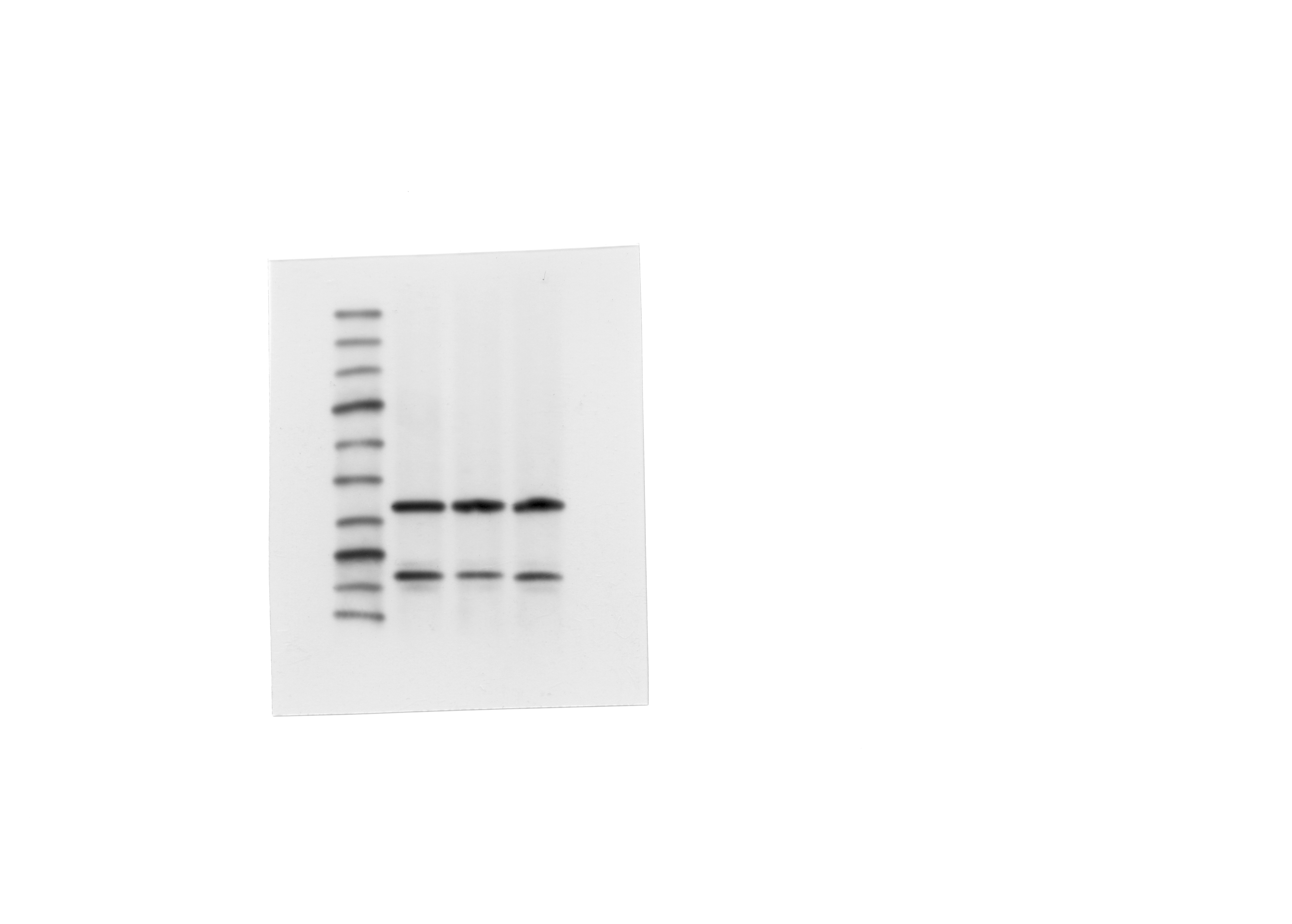

Supplement: Supplementary Table 3 — Gels and Blots image. [file DataSheet3.zip › Supplementary3 Gels and Blots image/Fig3B.Claudin-1∩╝ê1∩╝ë.tif]
